# Amphiphilic Dendritic Hydrogels with Carbosilane Nanodomains: Preparation and Characterization as Drug Delivery Systems

**DOI:** 10.1021/acs.chemmater.2c03436

**Published:** 2023-03-22

**Authors:** Judith Recio-Ruiz, Riccardo Carloni, Srivathsan Ranganathan, Laura Muñoz-Moreno, María José Carmena, Maria Francesca Ottaviani, Francisco Javier de la Mata, Sandra García-Gallego

**Affiliations:** † University of Alcala, Department of Organic and Inorganic Chemistry and Research Institute in Chemistry “Andrés M. Del Río” (IQAR), 28805 Madrid, Spain; ‡ Cancer Early Detection Advanced Research Center (CEDAR), Oregon Health and Science University, Knight Cancer Research Building, 2720 S Moody Avenue, Portland, Oregon 97201, United States; § Department of Systems Biology, University of Alcala, 28805 Madrid, Spain; ∥ Department of Pure and Applied Sciences, University of Urbino “Carlo Bo”, Urbino 61029, Italy; ⊥ Networking Research Center on Bioengineering, Biomaterials and Nanomedicine (CIBER-BBN), 28029 Madrid, Spain; # Institute Ramón y Cajal for Health Research (IRYCIS), 28034 Madrid, Spain

## Abstract

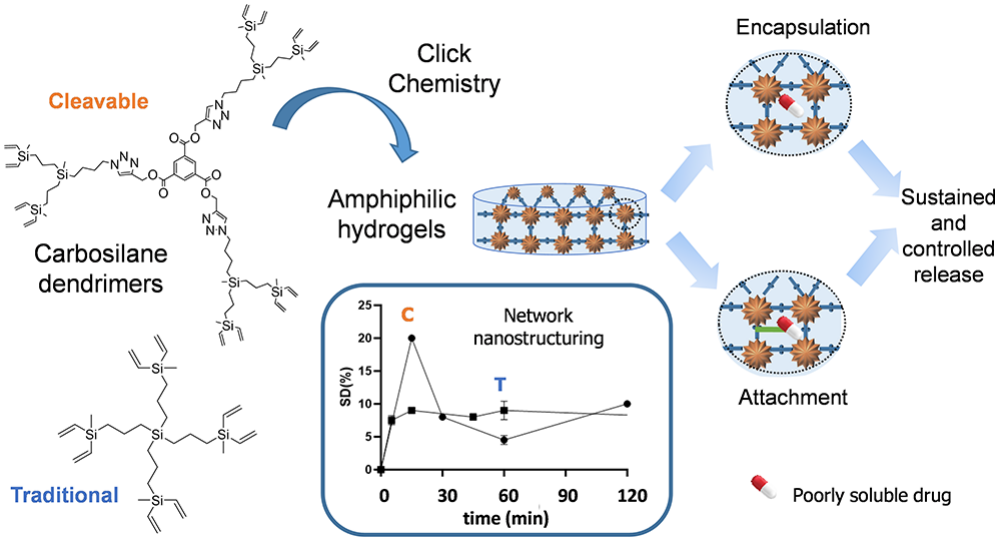

Carbosilane dendrimers are hyperbranched lipophilic scaffolds widely explored in biomedical applications. This work exploits, for the first time, the ability of these scaffolds to generate functional hydrogels with amphiphilic properties. The monodispersity and multivalency enable a precise synthetic control of the network, while the lipophilicity improves the compatibility with poorly soluble cargo. The first family of cleavable carbosilane dendrimers was designed for this purpose, overcoming one of the main drawbacks of these type of dendrimers. Biodegradable dendritic low-swelling hydrogels with aromatic nanodomains were easily prepared using the highly efficient click thiol–ene chemistry. Our studies through electron-paramagnetic resonance, molecular dynamics simulations, and experimental assays confirmed the impact of the carbosilane dendritic nanodomains in both the encapsulation and the release pattern of model drugs such as ibuprofen and curcumin. Curcumin-loaded hydrogels were further tested in *in vitro* assays against advanced prostate cancer cells. The dendritic hydrogels not only enabled drugs encapsulation; as proof of concept, ibuprofen was efficiently attached via fluoride-promoted esterification and was enzymatically cleaved, achieving a controlled release over time.

## 1 Introduction

Hydrogels are three-dimensional hydrophilic networks capable of absorbing water without dissolving.^1^ These materials are soft, flexible, and porous and present high water content, increasing their biocompatibility. Accordingly, they have found numerous uses in the production of contact lenses, hygiene products, and wound dressings as well as in drug delivery and tissue engineering applications.^2^ However, their inherent hydrophilic nature minimizes their compatibility with hydrophobic cargo, which represents 40% of marketed drugs and 90% of drugs under research.^3^

Another limitation of current hydrogels is the poor control over the network structure during the synthetic process.^4^ Physical hydrogels are formed by molecular entanglements and physical interactions, leading to reversible gels with poor mechanical properties, gelation time, and variable pore size. On the contrary, chemical hydrogels are based on chemical cross-linking, which generates highly stable materials with viscoelastic properties. The most common approaches toward these hydrogels are: (1) the 3D polymerization of a hydrophilic monomer using a multifunctional cross-linker, which requires extensive purifications to remove toxic residual monomers, or (2) the cross-linking of preformed hydrophilic polymers.^2^

Manipulating hydrogel structures at the nanometer level is a wise approach to tune their mechanical and physical properties. Composite hydrogels are generated by introducing different types of nanoparticles within the network, which can be dispersed or integrated in the hydrogel matrix, or they even act as cross-linkers of the network.^5^ In this sense, different examples have been described to improve compatibility with hydrophobic cargo. Cyclodextrins can form inclusion complexes and thus increase the solubility of certain compounds in water.^6^ Other examples are nanoemulsions, polymer nanoparticles, or micelles.^5^ Domains created by the self-assembly of the hydrophobic moieties in aqueous environments lead to nanostructured hydrogels as well as an improved loading of hydrophobic cargo.

Dendritic macromolecules have also emerged as interesting cross-linking agents in the synthesis of hydrogels. Their multivalent and multifunctional nature offers advantages both in the formulation and in the physico-chemical properties of these materials.^7,8^ Dendrimers are monodisperse, highly branched molecules, which enable a precise control on the design of the network. Different dendritic networks have been previously described in the literature, mainly based on dendritic polyesters^9^ or dendritic polyglycerols^10−12^ as cross-linking points. Some of the most relevant biomedical applications of these networks are antimicrobial therapy, soft- and hard-tissue patches, cell scaffolds, and drug delivery.

This work explores, for the first time, the use of carbosilane dendrimers in the design of hydrogels toward biomedical uses. Beyond the advantages offered by all dendrimers (monodispersity and multivalency, which translate into synthetic control), carbosilane scaffolds appear attractive due to their highly stable, inert, and lipophilic nature, which can increase the compatibility with lipophilic cargo as well as boost hydrogel nanostructuring. Carbosilane dendrimers have been broadly explored in the biomedical field, exhibiting unique activities as antiviral and antibacterial agents as well as a high efficiency to carry drugs or nucleic acids.^13,14^ Furthermore, Muzafarov et al. explored their ability to form networks by cross-linking the alkene-decorated dendrimer G6(all)_256_ with the smaller dendrimer G2(H)_12_ or with tetramethyldisiloxane, both acting as difunctional cross-linkers due to the steric hindrance of the sixth-generation dendrimer.^15^ Nevertheless, the non-degradable properties of carbosilane scaffolds can be a problem in the pharmaceutical field.

Herein, a family of new cleavable carbosilane dendrimers is designed and evaluated as cross-linkers in the synthesis of rigid hydrogel networks. The characterization of the hydrogels and their evaluation as drug delivery agents revealed important differences arising from the different nanodomains created by the carbosilane moieties, as it will be thoroughly described.

## 2 Experimental Section

Comprehensive details of the materials and methods used in this work are described in the Supporting Information. Synthetic protocols for compounds **1–5** and hydrogels **H3–8** are described below. The structure and purity of **1–5** were confirmed via ^1^H, ^13^C, 2D-NMR, and MALDI-TOF using a Bruker Neo400 spectrometer, a Bruker Ultraflex TOF/TOF spectrometer, and elemental analysis. Hydrogels were characterized through their swelling degree (SD %), cross-linking degree (CD %), and RAMAN-confocal microscopy (Thermo Scientific DXR). The loading and release of cargo from the hydrogels were explored via electron paramagnetic resonance (EPR) (Bruker EMX spectrometer), HPLC (Agilent 1200), and MD simulations (GROMACS-2018). *In vitro* cytotoxicity was measured through MTT assays on PC3 prostate cancer cells.

### 2.1 Synthesis and Characterization of Carbosilane Dendrimers

#### 2.1.1 Compounds **1** and **2**

The one-pot fluoride-promoted esterification (FPE) protocol was employed.^16^ Trimesic acid (TMA) (0.5 g, 2.38 mmol) was slowly added over a suspension of CDI (1.16 g, 7.15 mmol) in EtOAc (2 M), while heating the mixture at 50 °C for 30 min. The tri-imidazolyde derivative **1** was not isolated. Subsequently, CsF (0.2 equiv/OH) (0.26 mg, 0.17 mmol) and propargyl alcohol (1.2 equiv/COOH) (0.48 g, 8.57 mmol) were added, and the reaction proceeded at 50 °C. Upon completion after 4 h, the mixture was brought to r.t., and excess imidazolyde-activated acid was quenched by washing with 10% NaHCO_3_ (3 × 100 mL) and brine (3 × 100 mL), dried over anhydrous MgSO_4_, filtered, and evaporated to dryness. Compound **2** was isolated as a white solid (0.5 g, 65%). ^1^H and ^13^C NMR shifts were in agreement with those previously reported.^17^

#### 2.1.2 General Procedure for ArGnV_*m*_ dendrimers

Compound **2** and dendron N_3_GnV_*m*_^18^ (1 equiv/alkyne) were dissolved in the minimum amount of THF. An aqueous solution of NaAsc (0.3 equiv/N_3_) and another of CuSO_4_ (0.15 equiv/N_3_) were added, maintaining a 10:1 THF/H_2_O ratio. The mixture was stirred for 24 h at r.t., and then, the dendrimer was extracted in AcOEt, using a small amount of EDTA in the aqueous phase to improve Cu(II) removal. The organic phase was dried over anhydrous MgSO_4_, filtered, and evaporated to dryness.

#### 2.1.3 ArG1V_6_ (**3**)

Dendrimer **3** was synthesized through the general procedure, using the following reagents: compound **2** (55.3 mg, 0.171 mmol), N_3_G1V_2_ (**I**) (100 mg, 0.512 mmol), CuSO_4_ (19.2 mg, 0.0768 mmol), and NaAsc (30.5 mg, 0.154 mmol).

^1^H NMR (400 MHz, CDCl_3_): δ 8.81 (s, 3H, Ar–*H*^Ph^), 7.66 (s, 3H, Ar–*H*^triazole^), 6.07 (m, 6H, −SiC*H*=CH_2_), 5.99&5.69 (m, 6H, −SiCH=C*H*_*2*_*)*, 5.48 (s, 6H, −COOC*H*_*2*_−), 4.34 (t, 6H, NC*H*_*2*_−), 1.93 (m, 6H, NCH_2_C*H*_*2*_−), 1.36 (m, 6H, −C*H*_*2*_CH_2_Si−), 0.66 (t, 6H, −C*H*_*2*_Si−), 0.07 (s, 9H, −SiC*H*_*3*_), ^13^C NMR (400 MHz, CDCl_3_): δ 164.8 (*C*OO), 142.3 (*C*^triazol^^e^), 136.4 (−Si*C*H=CH_2_), 135.2 (Ar–*C*H^Ph^), 133.4 (−SiCH=*C*H_2_), 131.1 (Ar–*C*^Ph^), 124.1 (*C*H^triazole^), 58.8 (−COO*C*H_2_−), 50.2 (N–*C*H_2_), 33.8 (NCH_2_*C*H_2_), 21.0 (−*C*H_2_CH_2_Si−), 13.6 (−*C*H_2_Si−), −5.29 (−Si*C*H_3_), C_45_H_63_N_9_O_6_Si_3_ (910.3 g/mol), Calcd % C, 59.37; % H, 6.98; % N, 13.85. Exp. % C, 59.22; % H, 7.21; % N, 12.88, *m*/*z*: predicted: *M* = 909.42, observed: M + Na^+^ = 932.4.

#### 2.1.4 ArG2V_12_ (**4**)

Dendrimer **4** was synthesized through the general procedure, using the following reagents: compound **2** (25.7 mg, 0.0793 mmol), N_3_G2V_4_ (**II**) (100 mg, 0.238 mmol), CuSO_4_ (8.91 mg, 0.0357 mmol), and NaAsc (14.1 mg, 0.714 mmol).

^1^H NMR (400 MHz, CDCl_3_): δ 8.81 (s, 3H, Ar–*H*^Ph^), 7.66 (s, 3H, Ar–*H*^triazole^), 6.10 (m, 12H, −SiC*H*=CH_2_), 5.99&5.69 (m, 12H, −SiCH=C*H*_2_), 5.47 (s, 6H, −COOC*H*_*2*_−), 4.33 (t, 6H, NC*H*_*2*_−), 1.91 (m, 6H, NCH_2_C*H*_*2*_−), 1.30 (m, 18H, −C*H*_*2*_CH_2_Si−), 0.60–0.50 (2m, 30H, −C*H*_*2*_Si−), 0.10 (s, 18H, −SiC*H*_3_), −0.12 (s, 9H, −SiC*H*_3_). ^13^C NMR (400 MHz, CDCl_3_): δ 164.8 (*C*OO), 138.8 (*C*^triazole^), 137.2 (−Si*C*H=CH_2_), 135.2 (Ar–*C*H^Ph^), 132.8 (−SiCH=*C*H_2_), 132.1 (Ar–*C*^Ph^), 124.1 (*C*H^triazole^), 58.8 (−COO*C*H_2_−), 50.3 (N–*C*H_2_), 34.2 (NCH_2_*C*H_2_), 22.0 (−NCH_2_CH_2_CH_2_*C*H_2_Si−), 18.4 (−*C*H_2_*C*H_2_Si−), 13.6 (−NCH_2_CH_2_*C*H_2_−), −5.09 (−Si*C*H_3_). C_81_H_135_N_9_O_6_Si_9_ (1583.8 g/mol). Calcd % C, 61.43; % H, 8.59; % N, 7.96. Exp. % C, 61.23; % H, 8.48; % N, 6.88, *m*/*z*: predicted: *M* = 1582.85; observed: M + Na^+^ = 1605.8.

#### 2.1.5 ArG3V_24_ (**5**)

Dendrimer **5** was synthesized through the general procedure, using the following reagents: compound **2** (12.4 mg, 0.0383 mmol), N_3_G3V_8_ (**III**) (100 mg, 0.115 mmol), CuSO_4_ (4.31 mg, 0.0172 mmol), and NaAsc (6.83 mg, 0.0345 mmol).

^1^H NMR (400 MHz, CDCl_3_): δ 8.81 (s, 3H, Ar–*H*^Ph^), 7.66 (s, 3H, Ar–*H*^triazole^), 6.12 (m, 24H, −SiC*H*=CH_2_), 5.99&5.69 (m, 36H, −SiCH=C*H*_*2*_*)*, 5.47 (s, 6H, −COOC*H*_2_−), 4.33 (t, 6H, NC*H*_2_−), 1.92 (m, 6H, NCH_2_C*H*_2_−), 1.33 (m, 42H, −C*H*_*2*_CH_2_Si−), 0.60–0.50 (m, 78H, −C*H*_*2*_Si−), 0.11 (s, 36H, −SiC*H*_3_), −0.11 (s, 18, −SiC*H*_3_). ^13^C NMR (400 MHz, CDCl_3_): δ 164.8 (*C*OO), 142.3 (*C*^triazole^), 137.3 (−Si*C*H=CH_2_), 135.2 (Ar–*C*H^Ph^), 132.8 (−SiCH=*C*H_2_), 131.1 (Ar–*C*^Ph^), 124.1 (*C*H^triazole^), 58.8 (−COO*C*H_2_−), 50.3 (N–*C*H_2_), 34.3 (NCH_2_*C*H_2_), 22.0 (−NCH_2_CH_2_CH_2_*C*H_2_Si−), 18.4 (−*C*H_2_*C*H_2_Si−), 13.6 (−NCH_2_CH_2_*C*H_2_−), −4.8 & −5.0 (−Si*C*H_3_). C_153_H_279_N_9_O_6_Si_21_ (2930.8 g/mol). Calcd % C, 62.70; % H, 9.60; % N, 4.30. Exp. % C, 62.03; % H, 9.65; % N, 3.95.

### 2.2 Synthetic Procedure for Dendritic Hydrogels

The selected dendrimer was dissolved in the minimum amount of THF/MeOH (1:2), and the mixture was degassed with argon. DTT (SH/ene 1:1) and DMPA (5% mol/alkene) were added. The reaction mixture was stirred gently until complete dissolution of the reagents and then exposed to UV light (365 nm, 30 W). After cross-linking, the gel was removed from the lamp, dried under vacuum, and weighed. The hydrogels were purified by washing with acetone under orbital shaking until complete removal of DMPA was observed by TLC.

### 2.3 Drug Loading and Release Assays in Dendritic Hydrogels

#### 2.3.1 Drug Encapsulation

The selected hydrogel was immersed in 1 mL of a saturated solution of the drug (ibuprofen or curcumin) in ethanol and exposed for 30 min at r.t. under light orbital shaking. Then, the gel was removed from the vial and dried under vacuum.

#### 2.3.2 Covalent Attachment of Ibuprofen

FPE protocol was employed.^19^ In the first step, ibuprofen (20 mg, 1.5 equiv/OH gel) was activated as imidazolyde by reaction with CDI (15.7 mg, 1.5 equiv/OH gel) in dry ethyl acetate, for 20 min at 50 °C and with stirring. After complete activation was confirmed by ^1^H NMR, CsF was added to the solution (3 mg, 0.2 equiv/OH) and then the gel (10.7 mg) was immersed in the solution. The esterification proceeded for 20 h at 35 °C with gentle stirring. Afterward, the gel was removed from the vial and washed with acetone on an orbital shaker until no by-product was detected by TLC.

#### 2.3.3 Enzyme-Promoted Ester Cleavage

The selected hydrogel (10.0 mg of pristine hydrogel **H3**; 14.0 mg of ibuprofen-bonded hydrogel **H3**; 7.6 mg of ibuprofen-encapsulated **H4**) was immersed in 500 μL of water solution comprising 20% fetal bovine serum (FBS) and stirred under orbital shaking at 37 °C. Samples (50 μL) from the solution were taken over time. HPLC was then employed to study the release of TMA (to quantify network degradation) or ibuprofen (to quantify drug release), as detailed in the Supporting Information.

### 2.4 Cytotoxicity Assays with Pristine and Curcumin-Loaded Dendritic Hydrogels

Cytotoxicity was evaluated on the prostate cancer cell line PC-3. Details about the hydrogel sterilization, CUR loading, and cytotoxicity assays of the hydrogels are included in the Supporting Information. For pristine hydrogels **H3–H8**, leach-out tests confirmed that no toxic substances were released from the gels. The cytotoxic effect on the PC3 of curcumin release was evaluated through the leach-out test (“dynamic conditions”) and through transwell assays (“static conditions”), at different exposure times. MTT assay was used to quantify the cytotoxic effect.

## 3 Results and Discussion

### 3.1 Synthesis and Characterization of Cleavable Carbosilane Dendrimers

The dendritic scaffold in carbosilane systems is highly stable due to the strength and low polarity of the Si–C bonds, but it is usually non-biodegradable. In order to improve its applicability in biomedical settings, we herein targeted the design of new carbosilane dendrimers with improved features: degradability and affinity toward aromatic drugs. Ester bonds in the scaffold are potential cleavable points, while the aromatic rings drive the hydrogel nanostructuring and increase the affinity toward lipophilic drugs through π–π and hydrophobic interactions. For this purpose, TMA was selected as the dendrimer core, given its ability to form strong intermolecular interactions.

**Figure 1:**
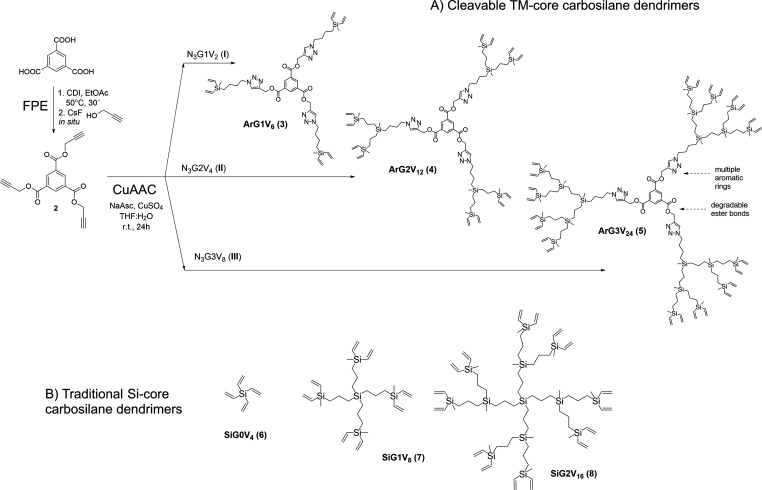
Carbosilane dendrimers used as cross-linkers in the preparation of amphiphilic hydrogels. (A) Synthetic route toward cleavable TM-core carbosilane dendrimers, based on FPE and CuAAC reactions. (B) Traditional Si-core carbosilane dendrimers.

The synthesis of the cleavable carbosilane dendrimers was accomplished through a two-step convergent approach relying on two orthogonal reactions: FPE and Cu-catalyzed azide–alkyne cycloaddition (CuAAC) (Figure 1A). Both reactions are outstanding tools for the synthesis of dendritic scaffolds due to their high efficiency, easy work-up, robustness, and versatility.^16,19^ The dendrimers’ core was prepared using a one-pot FPE protocol.^16^ In brief, TMA was activated with CDI for 30 min at 50 °C, generating the tri-imidazolyde-activated compound **1**, and subsequently reacted in situ with propargyl alcohol for 4 h at 50 °C in the presence of the CsF catalyst. After a simple work-up through several washing cycles, core **2** was obtained with 90% yield. The aromatic core **2** was then coupled through the CuAAC click reaction with vinyl-decorated carbosilane dendrons comprising azide groups in the focal point: N_3_G1V_2_ (**I**), N_3_G2V_4_ (**II**), and N_3_G3V_8_ (**III**). The dendritic precursors **I–III** were synthesized as previously reported.^18^

In this step, core **2** and the corresponding dendron were dissolved in the minimum amount of THF. Then, water solutions of sodium ascorbate and CuSO_4_ were added, generating in situ the Cu(I) catalyst. After 24 h at r.t., the final products were purified through washing and size-exclusion chromatography. Trimesic-core dendrimers ArG1V_6_ (**3**), ArG2V_12_ (**4**), and ArG3V_24_ (**5**) were isolated with 65% yield. Dendrimers **3–5** are soluble in common organic solvents such as CH_2_Cl_2_ or EtOAc but insoluble in water.

All the reactions were monitored by ^1^H and ^13^C NMR spectroscopy. The completion of the first step was confirmed by the appearance of a singlet at δ 8.9 ppm corresponding to the aromatic protons, a doublet at δ 4.9 ppm from the methylene group, and a triplet at δ 2.5 ppm corresponding to the alkyne proton. In ^13^C NMR spectra, a signal at 165 ppm confirmed the ester formation. The completion of the coupling step was observed by the disappearance of the signal at δ 2.5 ppm of the alkyne moieties and the appearance of the signal at δ 7.6 ppm assigned to the new triazole rings. In ^13^C NMR spectra, two peaks around 142 and 124 ppm confirmed the triazol formation. Bidimensional ^1^H–^13^C HSQC experiments were employed to confirm signal assignment. NMR spectra for all new dendrimers are included in the Supporting Information (Figures S1–S6). Due to the selectivity of the Cu(I) catalysis, only 1,4-disubstituted triazoles were obtained,^20^ thus keeping the highly desired monodispersity of the dendritic structures.

**Figure 2:**
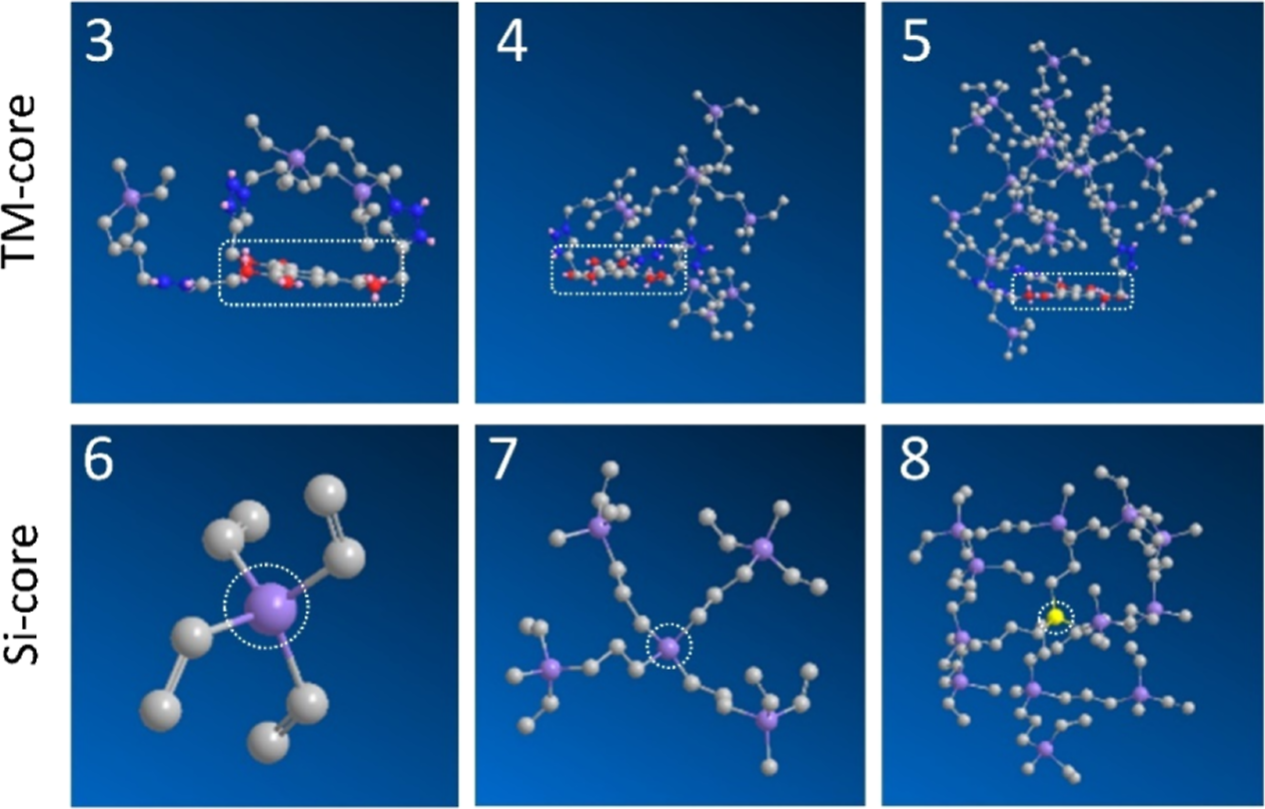
Snapshots from 3D spatial arrangement of carbosilane dendrimers **3–8** after MD job. The dendrimer core is highlighted. [Chem3D software: job 1 (minimize energy to minimum RMS gradient of 0.010) + Job 2 (MD. Step interval: 2.0 fs. Frame interval: 10 fs. Terminate after: 10,000 steps. heating/cooling rate: 1.000 kcal/atom/ps. Target temperature: 300 K)].

The multivalency and perfection of carbosilane dendrimers can be employed to accurately design dendritic networks. The precise control on the cross-linker size and number of reaction sites is a valuable tool. The spatial arrangement could also affect the network formation. This arrangement was studied employing the PerkinElmer Chem3D tool (v. 20.0.0.41). The molecule energy was minimized and then a preliminary MD job was run. The behavior of TM-core dendrimers **3–5** was compared to the one with silicon-core dendrimers SiG_*n*_V_*m*_**6–8** (Figure 2). For dendrimers **3–5**, the snapshots showed a planar arrangement of the dendritic core, which included the phenyl ring and the ester bonds, and a cage-like orientation of the carbosilane branches. A similar behavior was found for all three dendritic generations. Although the covalent cross-linking modifies the 3D disposition of the dendrimers, this “caging” effect could aid the entrapment of the drugs in the hydrogel pores. Silicon-core dendrimers showed a more uniform 3D arrangement driven by the tetravalent core, with the branches localized on all the directions. The 3D spatial arrangement of each dendrimer probably affects the cross-linking efficiency as well as drug loading, as it will be later explored.

### 3.2 Synthesis and Characterization of Dendritic Hydrogels

**Scheme 1:**
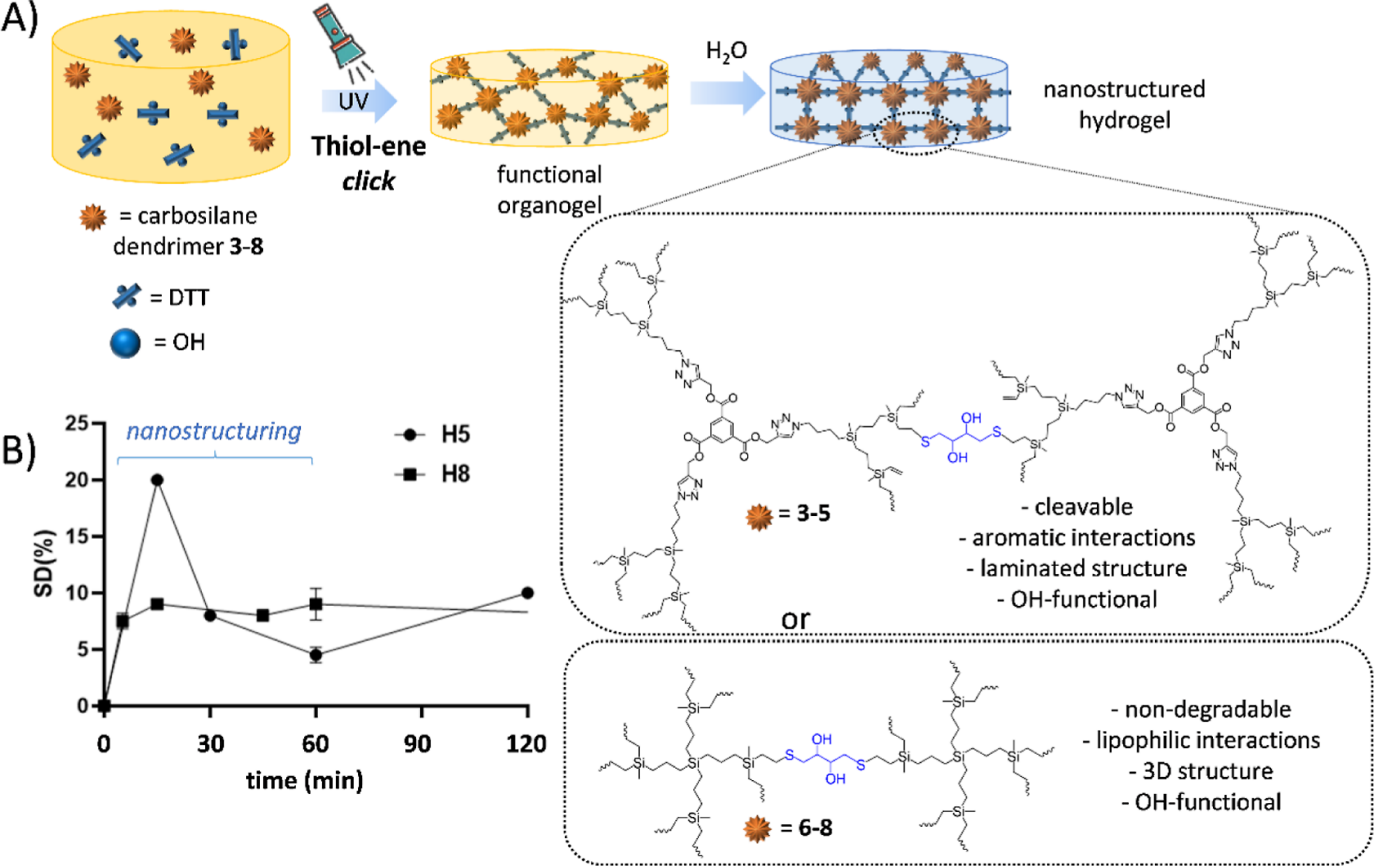
(A) Preparation of Dendritic Hydrogels through UV-Initiated Thiol–Ene Chemistry; in the Water Solution, the Nature (Type, Generation) of Carbosilane Dendrimer Drives the Hydrogel Nanostructuring; (B) Change in Swelling Degree (SD) during the First 2 h for Selected Hydrogels **H5** and **H8**, Exemplifying the Different Nanostructuring in Aromatic and Non-Aromatic Gels

The ability of vinyl-decorated carbosilane dendrimers **3–8** to act as cross-linkers and create dendritic networks was tested using the hydrophilic monomer dithiothreitol (DTT). DTT was selected due to its tetrafunctional nature: the two thiol groups are available for cross-linking, and the two hydroxyl groups can be used for post-functionalization purposes. The dendrimers and DTT were reacted via the highly efficient UV-initiated thiol–ene reaction (Scheme 1A). This click reaction has previously been employed in the design of networks and hydrogels, with outstanding results.^21,22^ In a general preparation, the dendrimer and DTT were dissolved in THF/MeOH mixture, using stoichiometric ratios between thiol/ene groups. Then, DMPA was added (5 mol % alkene), and the mixture was exposed to UV light (365 nm, 30 W, 1.050 μW/cm^2^). After cross-linking, the network was washed with acetone to remove DMPA and unreacted molecules and was dried under vacuum.

Several techniques offered insights into the structure and properties of the dendritic hydrogels. The cross-linking degree (CD %) indicates the efficiency of the cross-linking reaction, and the SD % represents the ability of absorbing water without dissolving. As Table 1 depicts, Si-core dendrimers favor a highly efficient network formation (CD > 95% in less than 1 h), which in turn decreases the swelling (SD < 12%). For these hydrogels, the swelling equilibrium is reached after 15 min in water (Figure S9, Scheme 1B). At short times (<2 h), the SD decreases as **H6** > **H8** > **H7**, probably related to the amphiphilic properties of the networks. At longer times, **H6** duplicates the SD for **H7**/**H8**, which exhibits no significant differences. The flexibility and 3D spatial arrangement of the dendrimers are probably behind this behavior, as predicted through MD simulations.

**Table 1:** Main Parameters from the Carbosilane Dendrimers Used in This Study and the Derived Dendritic Hydrogels

dendrimer	*M*_w_ (g/mol)	hydrogel	*m* % (D)^a^	reaction time (h)	CD (%)^b^	SD (%)^c^	ibuprofen loading^d^	curcumin loading^d^
ArG1V_6_ (**3**)	910.31	**H3**	66.3	3	70	16	7.3	6.0
ArG2V_12_ (**4**)	1583.79	**H4**	63.1	4	65	12	7.0	7.3
ArG3V_24_ (**5**)	2930.76	**H5**	61.3	4	65	8	7.2	6.6
SiG0V_4_ (**6**)	136.27	**H6**	30.6	1	95	12	6.0	2.7
SiG1V_8_ (**7**)	585.26	**H7**	48.7	1	96	6	5.5	5.0
SiG2V_16_ (**8**)	1483.23	**H8**	54.6	1	98	6	7.7	3.8

a Estimated mass % of dendrimer in the hydrogel.

b Cross-linking degree.

c Swelling degree in water.

d mg drug encapsulated/100 mg hydrogel.

On the contrary, TM-core dendrimers exhibit lower CD values (65–70%, after 3–4 h UV exposure) that slightly favor the swelling (SD 8–16%). Although all the generated networks can be considered low-swelling hydrogels,^23^ the swelling pattern is clearly different from that of Si-core dendrimers (Scheme 1B, Figure S8). During the first 15–30 min, the swelling reaches the maximum value, then abruptly decreases at 1 h, and finally increases in a more sustained rate. After 24 h, these hydrogels reach an equilibrium. It seems that the internal structure of these hydrogels requires longer times to readjust and accommodate the water molecules in the pores. A surprising “laminated” structure was also observed, which may be related to the planar disposition of the aromatic core and its potential “caging” effect. TMA forms strong intermolecular interactions, through H-bonding and ring stacking. For TM-core dendrimers, however, the only possible interaction is through ring stacking. TMA solvates with alcohols form tape structures, with an interlayer distance of 3.458 Å in EtOH.^24^ Depending on the solvent, there is a significant lateral shift of the layers to accommodate the packing of solvents in the ring structure of TMA. A similar behavior could explain the laminated nature of these hydrogels.

As expected from the components used, the characteristics of these hydrogels resemble those from “rigid networks”. For example, the networks formed from triallyltriazinetrione (TTT) and tris[2-(3-mercaptopropionyloxy)ethyl]isocyanurate (TMI) in a 1:1 ratio exhibit 9% SD in acetone and 99% CD.^25^ Employing an unbalanced ratio increases the SD and decreases the CD. Both trends are herein observed as well.

It is also worth highlighting that the estimated mass % of dendrimers in the hydrogel is kept approximately constant for TM-core dendrimers, while in the Si-core family, this value substantially increases from G0 (30%) to G2 (54%). This probably affects the behavior of each family, considering that the amphiphilic properties change in the same sense.

FT-RAMAN spectroscopy was used to further explore the efficiency of the cross-linking reaction. All networks were fed with a balanced stoichiometric SH/ene ratio, so we expected to see the disappearance of S–H and C=C stretching at 2564 and 1656 cm^–1^, respectively. **H7** was selected as a model hydrogel to perform a time-dependent study at 5, 20, and 60 min UV irradiation. We observed that we required at least 60 min reaction for the full consumption of reactive groups. For the aromatic hydrogels, the intrinsic fluorescence of the dendrimers hindered a correct detection of the signals, and this approach was uninformative. However, the lower CD % and NMR studies from the washing steps indicate the probable presence of some unreacted vinyl groups.

### 3.3 Impact of Aromatic Nanodomains in the Encapsulation of Low-Polarity Drugs: Experimental Assays and EPR Analysis

For the designed hydrogels, we expect an improved loading of low-polarity drugs due to (1) the presence of hydrophobic carbosilane nanodomains; (2) the multiple triazole and phenyl rings in **H3**, **H4**, and **H5**, which could increase the interaction with aromatic molecules; and (3) the pendant −OH groups that may assist in the loading process of certain drugs. In order to test these hypotheses, we selected two model aromatic compounds with low water solubility: ibuprofen (0.021 mg/mL), an anti-inflammatory drug, and curcumin (<0.1 mg/mL), a natural compound with potent antitumor, anti-inflammatory, and antioxidant properties.

#### 3.3.1 Encapsulation of Ibuprofen and Curcumin

When encapsulated, drugs are retained in the pores of the gel by non-covalent interactions, like electrostatic and hydrophobic interactions or π–π stacking. To maximize the drug loading, the hydrogel was immersed in a saturated solution of the compound in ethanol. After orbital shaking at room temperature, the hydrogel was removed from the solution and evaporated to dryness. The amount of non-absorbed drug in the ethanol solution was quantified by HPLC. The study revealed that only 30 min was required to reach the maximum loading of the drug, probably due to an efficient “sponge” effect of the hydrogel. For both drugs, we observed a higher loading in hydrogels with aromatic regions (Table 1), probably due to an increased interaction with the dendritic rings as well as higher accessibility from a lower cross-linking degree.

For the aromatic hydrogels **H3**–**H5**, a similar loading of ibuprofen was observed for all generations. The estimated mass percentage of dendrimers—and thus the amphiphilic properties—in these hydrogels is kept approximately constant for TM-core dendrimers, which could explain the comparable π–π interactions between the networks and the drug. However, a different behavior was observed for curcumin. In this case, **H4** was the most efficient, indicating that other parameters beyond π–π interactions may be involved, like the pores size or the internal arrangement of the nanodomains. For Si-core hydrogels **H6** and **H8**, it is worth highlighting that the amount of loaded ibuprofen duplicates that of curcumin, probably arising from a smaller size of the ibuprofen which accommodates better in such densely packed networks.

#### 3.3.2 EPR Evaluation Using the 4-Benzoyloxy-TEMPO Probe

**Figure 3:**
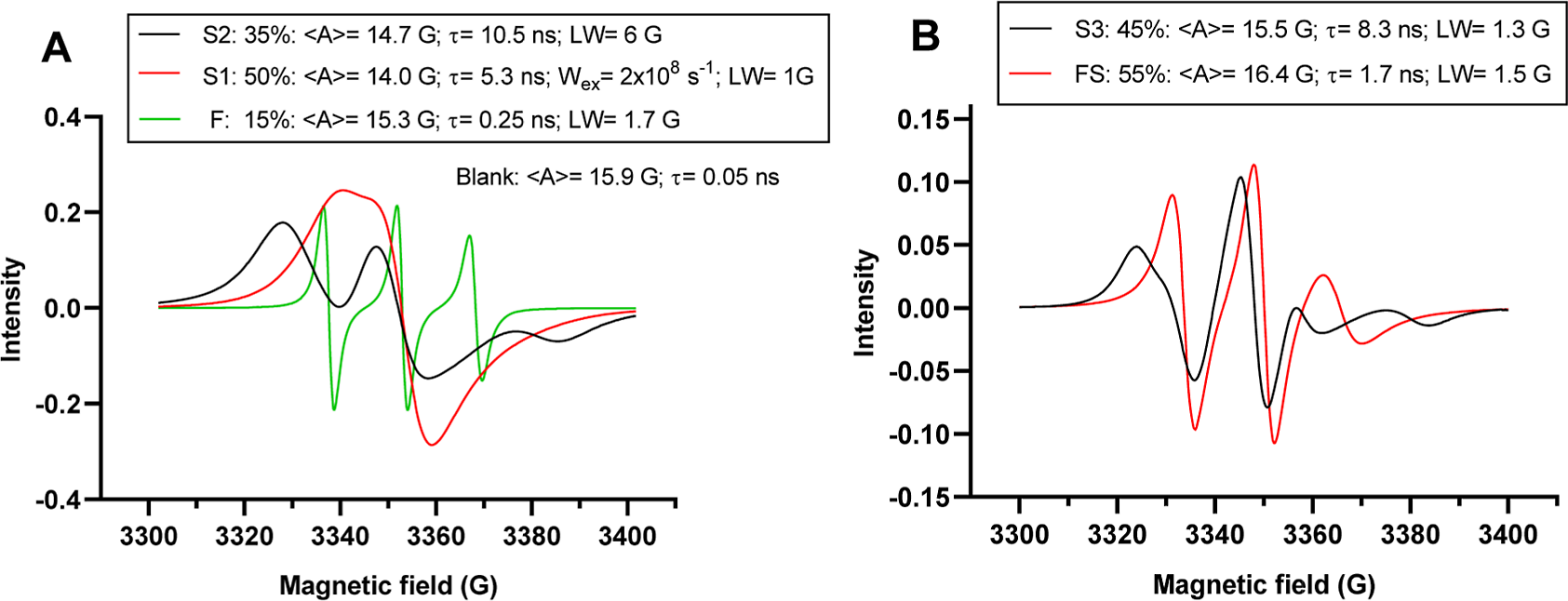
Computed spectra for the Bz-TEMPO probe adsorbed in aromatic hydrogels **H3** (A) and **H5** (B).

In order to gain further insights into the interactions responsible for the higher loading in aromatic hydrogels, we explored the loading of the probe 4-benzoyloxy-TEMPO through EPR. Spin-probe-aided EPR is an efficient technique to evaluate the mesh size of gel systems and to provide information on local interactions inside gels.^26^ In this work, aromatic hydrogels **H3** and **H5** were immersed into a saturated solution of the probe in ethanol and then studied through experimental and computer-aided EPR. Figures S9 and S10 show the experimental EPR spectra of the probe in ethanol solution adsorbed onto the hydrogel internal/external surface and their computation. Figure 3 presents the different components extracted from the computations of both hydrogels.

For **H3**, the computation needed the addition of three different spectral components indicated as F, S1, and S2 (Figure 3A). The legend reports the main parameters of computation, which are detailed in the Supporting Information. The analysis of the spectrum was performed by first computing the F (=Fast) component (green line in Figure 3A). This is at a quite low relative percentage (15%), but the spectral features are easily recognized since the F component is constituted by 3 well-resolved hyperfine lines, as usually found for fast moving probes. Computation of the F component was carried out using parameters indicative of a medium-low polarity (⟨*A*⟩= 15.3 G) and a fast mobility (τ = 0.25 ns). Interestingly, these parameters were different with respect to those found for the non-adsorbed (blank) solution (⟨*A*⟩= 15.9 G; τ = 0.05 ns). The lower ⟨*A*⟩ and higher τ values for the fast-moving probes in the adsorbed solution with respect to the non-adsorbed one suggest that the hydrogel surface affects the fast-moving probes, decreasing both polarity and mobility.

After subtracting this computed F component, the spectrum was clearly constituted by two “Slow” components. One of them was responsible of the two “shoulders” appearing at low and high fields, which allowed us to compute the S2 component. Indeed, these shoulders were due to the resolution of the anisotropic magnetic components, mainly the *A*_*ii*_ components, arising from the slowing down of mobility. The τ values significantly increases from the F component (0.25 ns) to the S1 component (10.5 ns) due to the fact that 35% of probes interact with the hydrogel surface. The decrease of micropolarity tested by the decrease of ⟨*A*⟩ from 15.3 to 14.7 G further supports the hypothesis of interactions at low polar sites. The line width also increases for the S2 component up to 6 G, which suggests that the probes sit at the hydrogel surface at quite close sites. Both parameters τ and *A* suggest a strong hydrogel–probe interaction, probably adjuvanted by the hydrophobic interaction between the phenyl substituent of 4-benzoyloxy-TEMPO and the benzylic rings of the internal structure of the hydrogel.

After subtraction of the computed S2 component, the last spectral component, termed S1, was extracted and computed. The S1 component contributed about 50% to the total spectrum and was computed using lower ⟨*A*⟩ and τ values (14.0 G and 5.3 ns, respectively) if compared to the S2 component. This means that most probes were entrapped into the hydrogel interstices. Such entrapment provoked a significant increase of the local concentration of the probes themselves that provoked a high exchange frequency, *W*_ex_, value of 2 × 10^8^ s^–1^.

A different situation was found for **H5** (Figure 3B): the spectral line shape is clearly different if compared to that found for **H3**. Interestingly, only two components contributed to the spectrum of **H5**. First, the fast component described for **H3** was no more present. Instead, a so-called FS component was identified, where FS indicates a condition in-between a fast and a slow motion regime, being τ = 1.7 ns. This FS component was 55% more abundant then the second component. The micropolarity also increased for **H5** compared to **H3**. This behavior was originally unexpected, given the large hydrophobic pockets that **H5** display in their interiors. Our hypothesis is that the G3-hydrogel cavities are large enough to host droplets of solvents, where benzoyloxy-TEMPO probes encounter a more polar environment compared to G1-hydrogels, leading to the origin of the FS component. The second component, indicated as S3 since the motion regime is slow (τ = 8.3 ns), also differs from the S1 and S2 components found for **H3**. The main difference was the absence of both line broadening (low LW) and exchange spin–spin effects (no *W*_ex_ needed in the computation). This effect is due to the more polar environment encountered by probes compared to **H3**. Also, the large **H5** interstices allowed a more homogenous and less packed distribution of the probes, explaining the absence of spin–spin interactions and line broadening in the spectra.

In summary, the dendrimer generation plays a crucial role in hydrogel nanostructuring as well as in drug loading.

### 3.4 Release Studies of Encapsulated Drugs: Experimental Assays and Molecular Modeling

#### 3.4.1 Drug Release: Impact of pH and Temperature

Drug release was explored by immersing the drug-loaded hydrogel in water solution under orbital shaking and quantifying the cumulative release over time by HPLC.

**Figure 4:**
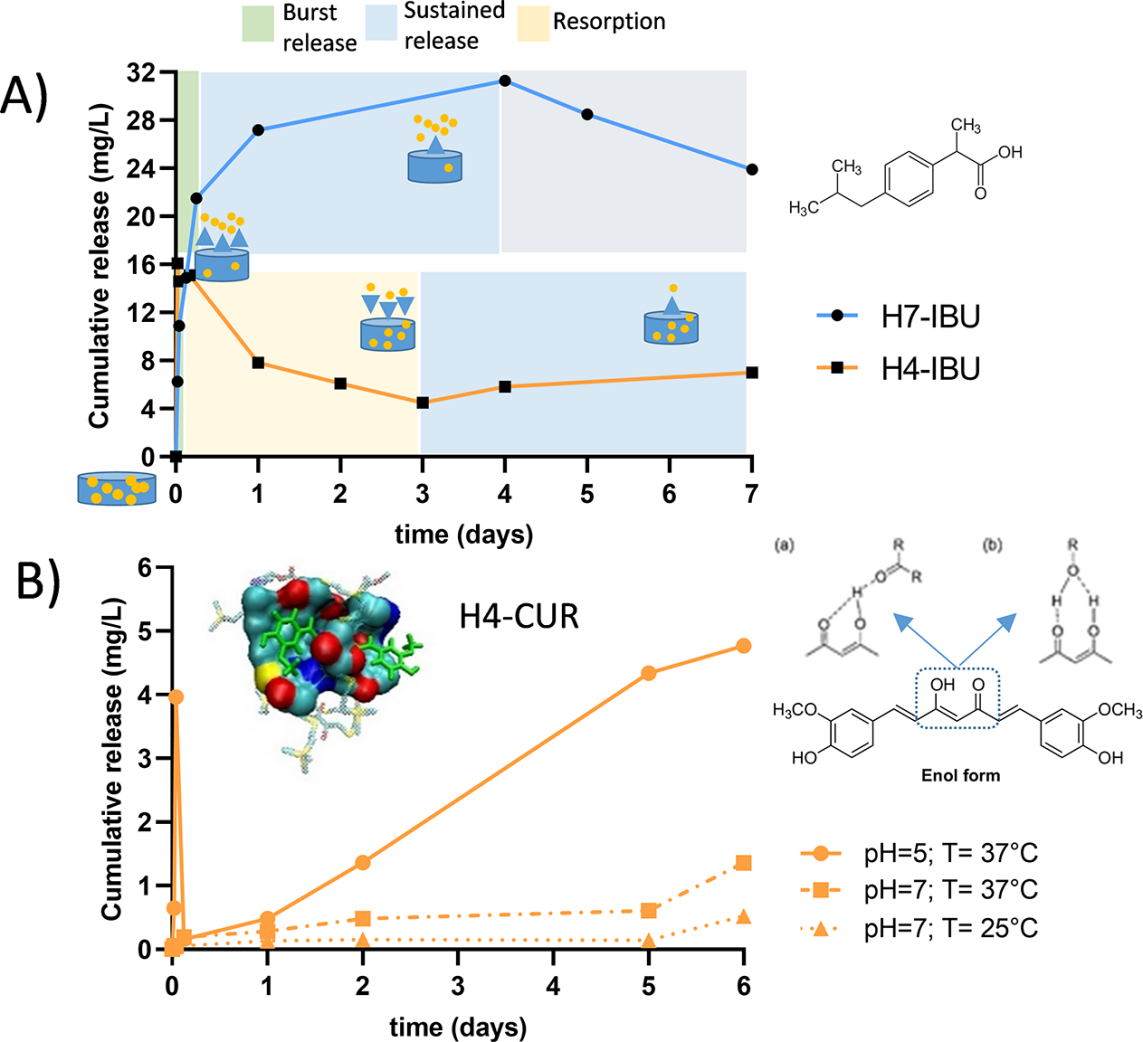
(A) Ibuprofen release curves from hydrogels **H4** and **H7** in distilled water at 25 °C. Different release zones are highlighted: burst (green), sustained (blue), resorption (yellow), and dilution effect (gray). B. Curcumin release curves from aromatic hydrogel **H4** at different temperatures and pH. The insert shows the computation of the interaction between curcumin (in green) and aromatic hydrogel **H3**. Close contacts are highlighted. The enol form of curcumin and potential H-bonding interactions with aromatic hydrogels are shown.

For ibuprofen, Si-core hydrogels produced a burst release in the first hours and a sustained release for 4 days (Figures 4A and S12). A quite different behavior was found for TM-core hydrogels. A potent burst release occurred in the first minutes, but then the drug concentration continuously decreased until day 3, when a sustained release started. An explanation could be found in the redistribution of the hydrogel nanostructure when immersed in water, with a potent “sponge effect” which later acted as a reservoir of the aromatic drug. In both cases, the drug release pattern resembles the swelling pattern (Figures S8 and S9), indicating a potential connection between both events.

Curcumin release was also evaluated. Si-core hydrogels show a sustained release of the cargo during the 40 days the experiment took place (Figure S13). However, and despite the higher loading of curcumin in aromatic hydrogels (>6 mg/100 mg), the release in water at 25 °C could not be quantified through HPLC, probably due to a strong interaction within the network arising from π–π stacking and H-bonding. To promote curcumin release from aromatic hydrogels, we changed the temperature and pH of the medium: from 25 to 37 °C, at pH 7.4 (in PBS buffer) and 5.0 (in citrate buffer). The results from curcumin-loaded **H4** are depicted in Figure 4B. Curcumin exists in different tautomeric (keto–enol) forms and assembles in aggregates in aqueous media. This equilibrium is significantly influenced by factors such as concentration, solution pH, and temperature.^27^ The *cis*-enolic form is the most stable in solution, while the keto formation is promoted at low pH or in protic solvents. At pH 7 and 25 °C, we observe a negligible release of curcumin. Under these conditions, curcumin molecules are probably dissociated or form small aggregates. They fit well in the hydrogel interstices and interact through π–π stacking and H-bonding as the computational studies indicate (Figure 4. B, insert). Increasing the temperature to 37 °C further dissociates the aggregates and destabilizes the interactions with the network, resulting in a slight increase in curcumin release. Nevertheless, it is the switch to pH 5 that produces a significant change. It has been described that, at 25 °C, the enolic form is favored in acidic solutions due to the more favorable non-covalent interactions (electrostatic or H-bonding) with the solvent and probably also with the hydrogel network (Figure 4B insert). However, at 37 °C, the keto–enol equilibrium becomes independent of pH, as the stabilizing interactions with the solvent become perturbed. In our case, the low pH promotes curcumin release, confirming a destabilization of the interactions of the drug in the hydrogel pores. Overall, this assay confirmed a selective release under physiological conditions of the tumor acidic microenvironment, which is an interesting property for an antitumor treatment.

#### 3.4.2 Molecular Dynamics

**Figure 5:**
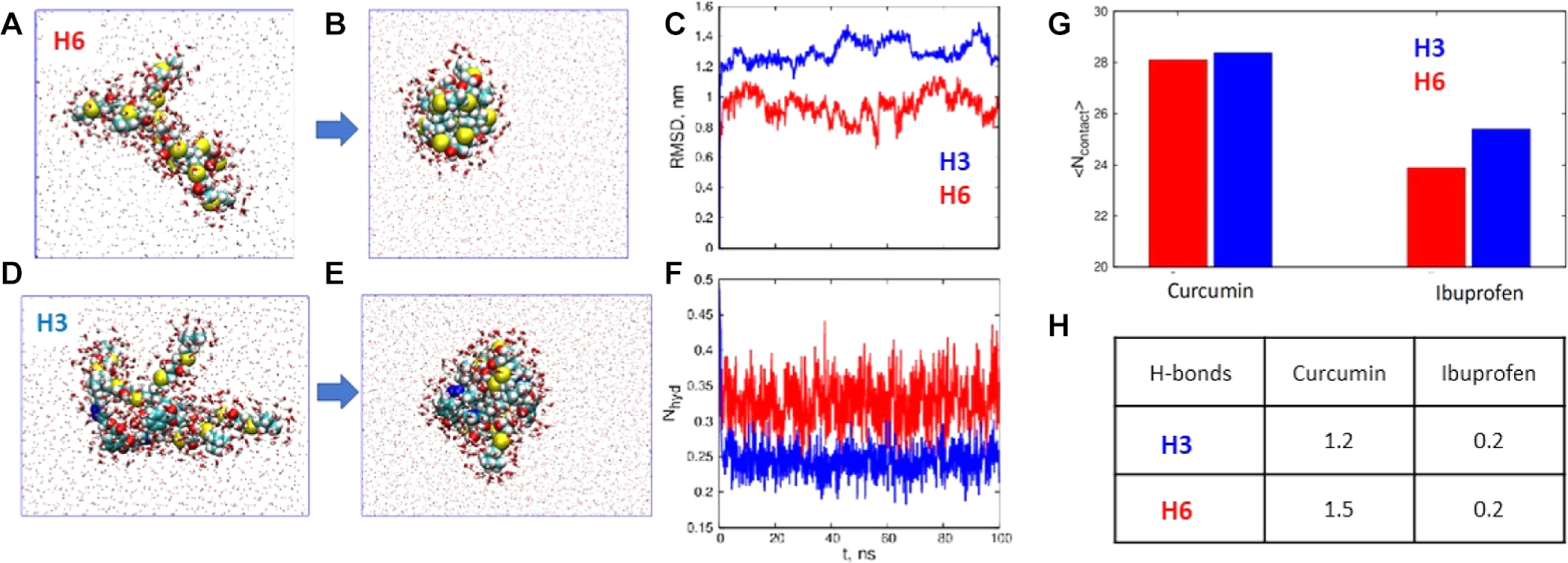
(A,B,D,E) Simulation snapshots of **H3** and **H6** in explicit water simulations. (C) Root mean square deviation of the hydrogels over the course of the simulation. (F) Normalized number of water molecules in the first hydration shell. (G) Normalized number of contacts between the drug molecule and the hydrogel. (H) Average number of hydrogen bonds.

To gain a molecular insight into the hydrogel nanostructuring in water and the interaction of the drugs within the hydrogel pores, MD studies were performed on selected hydrogels. Figure 5 depicts snapshots for cross-linking sections of hydrogels **H3** and **H6** (Figure S14). Figure 5C shows the root mean square deviations (rmsd) of the hydrogels, which exhibit a rapid change (collapse) of the structures followed by stability over the course of the simulation. According to Figure 5F, **H3** shows lower hydration than the non-aromatic **H6**; this could be explained by stronger interactions within the hydrogel network (like internal π–π stacking), which hamper the interaction with solvent molecules. This behavior is also in line with the EPR study, where we observed that third-generation **H5** presented large cavities capable of hosting droplets of solvents, while first-generation **H3** exhibited a higher packing of the probes in the interstices, which found a less polar environment. Interestingly, **H6** hydration also shows larger fluctuations, which could be indicative of higher flexibility of **H6** over **H3** (Figure 5F).

Both ibuprofen and curcumin show more contacts, and therefore a stronger interaction, with the aromatic hydrogel **H3** compared to **H6** (Figure 5G). Besides the H-bonding, especially with curcumin (Figure 5H), the aromatic **H3** system is able to do π–π stacking with both drugs, which supports the higher loading in this family of hydrogels.

#### 3.4.3 Proof of Concept: *In Vitro* Antitumor Assays

To explore the potential of these hydrogels as drug carriers in a more realistic situation, selected hydrogels were tested in *in vitro* assays against advanced prostate cancer cells PC3. Curcumin was selected considering the antitumor activity in PC3 cells (IC50 50 μM). Leaching out tests under dynamic conditions (orbital shaking) or static conditions (transwell plates) were used, and viability was analyzed through the MTT assay (see the Supporting Information for further details).

**Figure 6:**
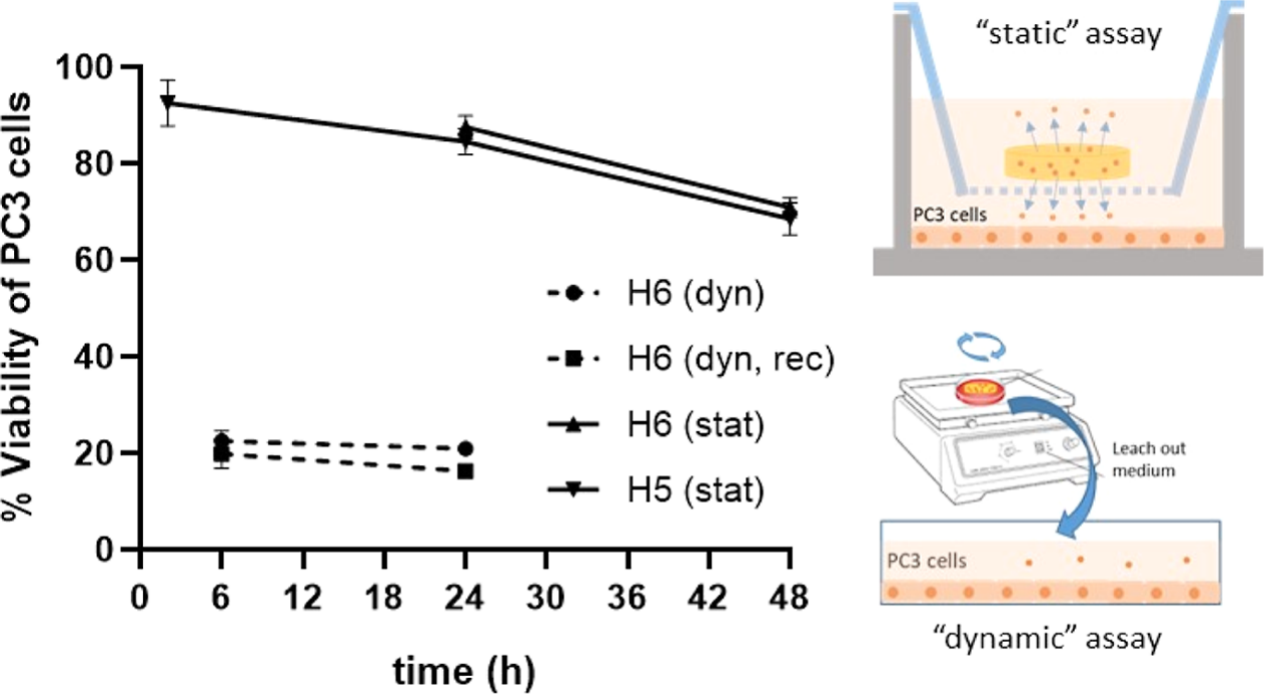
Viability of PC3 cells after curcumin release over time from selected hydrogels. In dynamic studies, cells were cultured with leach-out media from the hydrogels (33.7 mg) obtained under orbital shaking for 24 h. As control, non-treated cells were used (100% viability). In static studies, cells were exposed to in situ release from hydrogels (14.5 mg) using transwells. As control, pristine hydrogels were used (100% viability).

Pristine hydrogels **H3–H8** were immersed in cell culture medium for 24 h under orbital shaking. Then, PC3 cells were cultured with the extracted media and viability was analyzed. Results showed 100% cell viability (data not shown), confirming that no toxic products were released from the dendritic hydrogels. In a second assay, we studied curcumin release over time in “dynamic conditions”, extracting the drug under orbital shaking. The Si-core hydrogel **H6** was selected in this assay, which had exhibited the lowest loading. Employing hydrogel pieces of an average of 33.7 mg, we observed 77% cell death after 6 h exposure, which slightly increased to 79% after 24 h (Figure 6). It is worth highlighting that these hydrogels were reusable. After thorough cleaning and subsequent loading, the second-round assays showed 81 and 84% cell death at 6 and 24 h exposure, respectively. In a third assay, we performed curcumin release studies using transwell plates, providing information about the “static” release. The loaded hydrogels (14.5 mg) were located at the upper compartment, while PC3 cells were grown in the lower compartment. In this case, Si-core hydrogel **H6** and aromatic hydrogel **H5** were tested. Under such static conditions, aromatic hydrogel **H5** produced 9, 16, and 32% cell death after 2, 24, and 48 h, respectively, which are slightly better results than hydrogel **H6**. Overall, these results show the potential of the dendritic hydrogels as carriers of antitumor drugs as well as the impact of the assay conditions.

### 3.5 Covalent Attachment of the Drugs to the Hydrogels: Enzyme-Promoted Release and Network Degradation

#### 3.5.1 Drug Bonding through FPE

**Figure 7:**
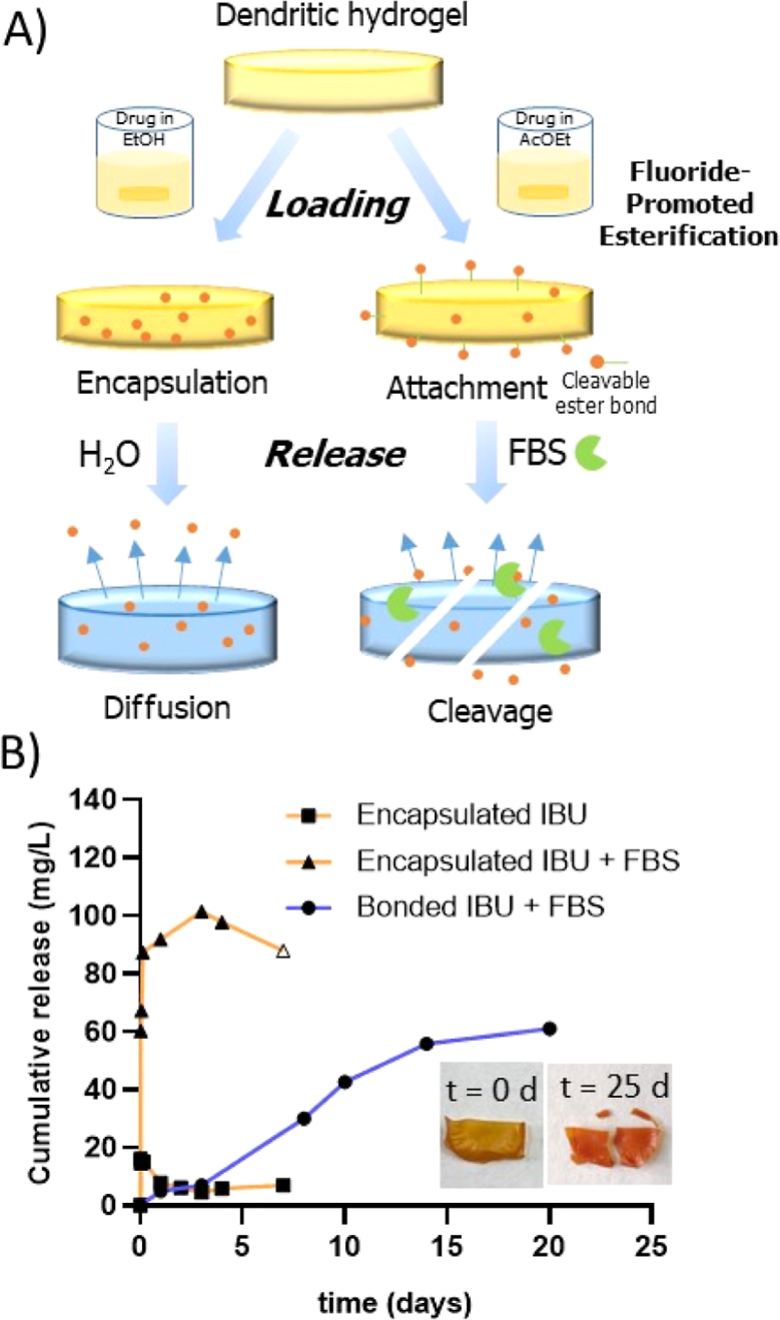
(A) The dendritic hydrogels are highly versatile in the cargo loading. The drug can be encapsulated and released through diffusion, or it can be covalently attached through cleavable ester bonds. (B) Example of cumulative release of ibuprofen encapsulated in **H4**, released in water (■) or in the presence of esterases (▲), or cleaved from **H3** after exposure to esterases (●). Symbol Δ indicates exclusive dilution effect. The insert shows the fractures in the hydrogel during the span of the experiment.

The versatility of the dendritic hydrogels was further explored using a post-functionalization strategy (Figure 7A).

In this second approach, the drug can be covalently bound to the network by degradable ester bonds, through the pendant hydroxyl groups. This enables a more precise control on the release of the drug. For this experiment, ibuprofen was selected due to the available −COOH group. FPE proved to be a highly useful approach for the esterification of molecules to solid materials due to the simple protocol and easy work-up. Ibuprofen was reacted with CDI for 20 min at 50 °C, and then, the hydrogels **H4** and **H6** were immersed in the solution, in the presence of the CsF catalyst, for 20 h, with continuous orbital shaking at 35 °C. Hydrogels were then removed from the solution and washed with water and acetone. After drying, we quantified the amount of attached drug through weight difference. For **H4**, 30 mg/100 mg hydrogel was attached; for **H6**, 60 mg/100 mg hydrogel was linked. This means 4 and 10 times higher than the encapsulated drug, respectively. The functionalized hydrogels were studied through FTIR spectroscopy, observing the decrease in the band at 3370 cm^–1^ assigned to the O–H stretching and the appearance of the 1735 cm^–1^ band due to the −COOR stretching (Figure S15).

#### 3.5.2 Enzyme-Promoted Drug Release and Network Degradation

The presence of ester bonds, both as a link between the drug and the hydrogel and in the cross-linking points of the network, provides cleavable points with two different purposes: the selective release of the drug and the degradation of the hydrogel in the presence of esterases. To confirm these hypotheses, selected hydrogels were exposed to water solutions containing 20% FBS under orbital shaking at 37 °C for several days. First, pristine hydrogel **H3** was tested for 12 days. Although the hydrogel was visually broken during this time, no TMA was detected by HPLC. This indicates that the hydrogel was progressively broken, but, over the tested time, the dendrimer core was not released, as three ester bonds must be simultaneously cleaved. In a second assay, IBU-encapsulated **H4** was exposed to the FBS solution; a progressive release of ibuprofen was observed during the first 4 days, much higher than the release in water (Figure 7B). This confirms the rupture of the hydrogel network by the esterases, which favored the release of the drug. A third assay was performed with IBU-loaded **H3**, where the release of ibuprofen and TMA was simultaneously tested for 25 days. In this case, a sustained release of ibuprofen was confirmed over the span of the experiment (Figure 7B), but again no TMA was detected. This study confirmed that covalent attachment of the drug is a more efficient approach than encapsulation, which enables a higher loading as well as a sustained release over a long period of time.

## 4 Conclusions

Carbosilane dendrimers are promising tools for the design and nanostructuring of dendritic hydrogels. The structural perfection, the multivalent nature, the lipophilicity, and the stability offer unprecedented control over the synthesis of the networks as well as over the drug loading and release. In particular, the new family of cleavable carbosilane dendrimers herein reported opens new avenues in the field of biomedicine, overcoming the non-degradability challenge as well as the tedious synthesis by employing orthogonal and highly efficient reactions such as CuAAC and FPE.

As we demonstrated, the nature of the carbosilane dendrimer produces an outstanding impact on the hydrogel properties and is responsible for the network nanostructuring. Both Si-core and TM-core dendrimers efficiently form networks through the thiol–ene chemistry, but the nature and generation of the dendritic cross-linker affect the cross-linking efficiency and swelling ability as well as the drug loading and release. The drug release pattern is surprisingly different between both families of hydrogels due to the adjustment of the nanodomains in water, and it also depends on the nature of the drug. For example, TM-core dendrimers strongly bind curcumin, and it is necessary to switch to pH 5 and 37 °C to generate a potent release. This pH-responsive behavior is relevant in cancer applications, producing a selective drug release in the tumor environment.

Finally, the versatility of these hydrogels was also exemplified through their ability to attach drugs through degradable bonds. We demonstrated, for the first time, that FPE is an outstanding tool to modify solid materials in an efficient and clean way. Hydrogels prepared using cleavable carbosilane dendrimers underwent degradation and drug release in the presence of esterases. This strategy enables a higher loading as well as a more controlled release of the drug.

Overall, dendritic hydrogels with carbosilane nanodomains appear as a tunable, versatile, and efficient approach to improve the loading and controlled release of drugs with poor water solubility. Furthermore, the biodegradable nature of the new cleavable carbosilane dendrimers and the derived hydrogels opens new avenues in a myriad of biomedical applications.

## Supporting Information Available

Detailed materials and methods; ^1^H, ^13^C, HSQC NMR, and MALDI-TOF spectra of dendrimers **3**–**8**; and characterization of hydrogels (swelling degree, EPR spectra, drug release curves, FTIR spectra) (PDF)
