# Supplementary material for: Amphiphilic Dendritic Hydrogels with Carbosilane Nanodomains: Preparation and Characterization as Drug Delivery Systems
Source: Chem Mater. 2023 Mar 22;35(7):2797–807. doi: 10.1021/acs.chemmater.2c03436 (PMC10101558; doi:10.1021/acs.chemmater.2c03436)
Supplement: Supplementary file 1 — cm2c03436_si_001.pdf [file cm2c03436_si_001.pdf]

Electronic Supporting Information

Amphiphilic dendritic hydrogels with carbosilane nanodomains: preparation and characterization as drug delivery systems

Judith Recio-Ruiz,[a] Riccardo Carloni,[b] Srivathsan Ranganathan,[b] Laura Muñoz-Moreno,[c] María José Carmena,[c] Maria Francesca Ottaviani,[d] Francisco Javier de la Mata [a,e,f] and Sandra García-Gallego* [a,e,f]

a. University of Alcala, Department of Organic and Inorganic Chemistry and Research Institute in Chemistry "Andrés M. Del Río" (IQAR), 28805, Madrid, Spain. judith.recio@edu.uah.es

b. Cancer Early Detection Advanced Research Center (CEDAR), Oregon Health and Science University, Knight Cancer Research Building, 2720 S Moody Ave, Portland, OR 97201

c. University of Alcala, Department of Systems Biology, 28805, Madrid, Spain.

d. Department of pure and applied Sciences, University of Urbino "Carlo Bo", Urbino, 61029, Italy.

e. Networking Research Center on Bioengineering, Biomaterials and Nanomedicine (CIBER-BBN), 28029, Madrid, Spain;

f. Institute Ramón y Cajal for Health Research (IRYCIS), 28034, Madrid, Spain.

Materials and methods……………………………………………………………………………………………………3

Figure S1. ^{1}H and ^{13}C NMR spectra of dendrimer ArG1V6 (**3**) ……………………………………………………6

Figure S2. ^{1}H-^{13}C HSQC spectrum of dendrimer ArG1V6 (**3**) …………………………………………………6

Figure S3. ^{1}H and ^{13}C NMR spectra of dendrimer ArG2V12 (**4**) ……………………………………………………7

Figure S4. ^{1}H-^{13}C HSQC spectrum of dendrimer ArG2V12 (**4**) …………………………………………………7

Figure S5. ^{1}H and ^{13}C spectra of dendrimer ArG3V24 (**5**) ……………………………………………………………8

Figure S6. ^{1}H-^{13}C HSQC spectrum of dendrimer ArG3V24 (**5**) …………………………………………………8

Figure S7. **H7** Hydrogel formation after 2, 5 and 10 min exposure to UV-light…………………………8

Figure S8. Swelling degree over time for aromatic-core hydrogels **H3, H4** and **H5**……………………9

Figure S9. Swelling degree over time for Si-core hydrogels **H6**, **H7** and **H8**………………………………9

Figure S10. Experimental and computed EPR spectra of **H3** with 4-Benzoyloxy-TEMPO …………9

Figure S11. Experimental and computed EPR spectra of **H5** with 4-Benzoyloxy-TEMPO ……….10

Figure S12. Ibuprofen-release curves for hydrogels **H4-H7**……………………………………………………10

Figure S13. Curcumin-release curves for hydrogels **H7-H8**…………………………………………………… 10

Figure S14. Selected crosslinking sections of **H3** and **H6** used in the MD simulations……………11

Figure S15. FTIR spectra of **H4**, before and after esterification of ibuprofen …………………………11

Figure S16. MALDI-TOF spectra of dendrimer **3** in DCTB……………………………………………………………12

Figure S17. MALDI-TOF spectra of dendrimer **4** in DCTB……………………………………………………………12

Figure S18. Captothecin-release curves from hydrogels **H4** and **H7**………………………………………13

Materials and methods.

Materials. Reagents and solvents were purchased from commercial sources and used as received. 1,3,5-Benzenetricarboxylic acid (98%) and 4-Bromo-1-butene (97%) were purchased from Alfa Aesar. N,N'-Carbonyldiimidazole was purchased from Fluorochem. Propargyl alcohol, Copper(II) sulfate pentahydrate, (+)-Sodium L-ascorbate, Ethylenediaminetetraacetic acid, Tetravinylsilane (95%), Dichloromethylsilane, Karstedt's Pt catalyst, 18-Crown-6, Sodium azide (>99,5%) and Ibuprofen were purchased from Sigma Aldrich. Cesium fluoride (99,9%) was purchased from Glentham Life Sciences. 2,2-dimethoxy-2-phenylacetophenone (DMPA) was purchased from Acros Organics. Dithiothreitol (>99%) was purchased from Apollo Scientific. Sodium iodide (>99%) was purchased from Fisher Chemical. Curcumin was purchased from TCI. Acetone, ethyl acetate, tetrahydrofuran and methanol were purchased from Sigma Aldrich with HPLC grade. Vinyl-decorated dendrons N_3GnV_m (**I-III**) were synthesized as previously reported.[1]

Cell line. *In vitro* studies were performed using PC-3 cell line, an advanced stage of androgen-independent prostate cancer. PC-3 cells were obtained from ATCC (American Type Culture Collection). The cell line was routinely grown in culture medium RPMI-1640 supplemented with 10% fetal bovine serum (FBS) and 1% antibiotic/antifungal mixture (penicillin, streptomycin and amphotericin B) at 37°C in a humidified environment with 5% CO_2. The medium was renewed every 2-3 days and, when the cells reached 70-80% confluence, they are detached with trypsin for amplification by flask seeding or for performing the evaluations.

Nuclear Magnetic Resonance (NMR) spectroscopy. NMR spectra were acquired at CAIQ-UAH, in a Bruker Neo400 instrument, at room temperature and using $CDCl_3$ as solvent. The chemical shifts are expressed in ppm using the solvent as internal reference, in ^1H NMR ($CDCl_3$ δ (H)=7.24 ppm) and in ^{13}C NMR ($CDCl_3$ δ (C) = 77.0 ppm). When necessary, signals assignments were performed using (^1H-^{13}C)-HSQC-2D-NMR experiments.

Elemental analysis. Quantitative analyses of C, H and N were performed at CAIQ-UAH, employing a LECO CHNS-932 microanalyser.

MALDI-TOF mass spectrometry. Mass spectra were recorded using a Bruker Ultraflex III (TOF/TOF) spectrometer at SIDI-UAM. Dendrimers were dissolved in acetone. Samples were prepared in DCTB matrix with NaI and then analyzed.

RAMAN-confocal microscopy. Raman spectra were recorded using a Thermo Scientific DXR Raman confocal microscope, controlled by Thermo Scientific OMNIC 8.3.103 software for dispersive Raman, kindly provided by CINQUIFOR-UAH research group. A laser emitting at 780 nm with 10 mW and a confocal slot size of 50 µm was used. The microscope was set at 10× magnification under bright field illumination. The Raman spectra of all samples were recorded in the range 3300-400 cm^{-1} and subsequently normalized between 0 and 1 to facilitate comparison.

High Performance Liquid Chromatography (HPLC). Drug release was quantified on an Agilent 1200 HPLC equipment at CAIQ-UAH, using an ACE Excel 5 column. For ibuprofen quantification (detected at 220 nm), a mobile phase of 0.2% trifluoroacetic acid and acetonitrile (30:70) was used, with an injection volume of 10 µL. For curcumin quantification (detected at 425 nm), a mobile phase of 0,1% phosphoric acid and acetonitrile (30:70) was used, with an injection volume of 10 µL. For trimesic acid

quantification, and a mobile phase of 0,1% phosphoric acid and acetonitrile (55:45) was used, with an injection volume of 10 µL.

Hydrogels curing. Hydrogels were cured from solutions of DTT, the corresponding vinyl-decorated dendrimer and DMPA. The three compounds were dissolved in a THF:MeOH (1:2) mixture and argon bubbled for a few seconds before exposing the mixture to UV light. A UV darkroom Vilber CN-15.LC was used, with a total intensity of 30 W at 365 nm. Hydrogels were irradiated for 1-3 h, then evaporated to dryness and purified through multiple washing steps with acetone until by-products are removed.

Swelling studies. Hydrogels were swelled by immersion in a distilled water bath for several days. All gels were tested in duplicate. The swelling degree is calculated with the following equation:

$$SD\% = ((W-W_D) / W) * 100$$

where SD% is the percentage of swelling, W is the weight of the swollen gel and W_D is the dry gel mass after purifications.

Crosslinking studies. The crosslinking degree (CD%) is calculated to evaluate the effectiveness of the hydrogels formation. Such value changes as a function of the reaction stoichiometry, the solvent used and the reaction time. Optimal conditions vary for each hydrogel, but they all have in common the washing cycles, and the solvent used (THF/MeOH in 1:2 ratio). All the measurements were performed twice. The crosslinking percentage was calculated using the following equation:

$$CD\% =1 - (M_{UV}-M_W /M_{UV}) * 100$$

where CR% is the crosslinking percentage, W_{UV} is the dry mass after UV and M_W is the dry mass after purifications.

Electron Paramagnetic Resonance (EPR) studies. EPR spectra were recorded with a Bruker EMX spectrometer equipped with a Bruker ER 041 X G microwave bridge and a continuous-flow liquid helium cryostat (ESR900) coupled to an Oxford Instruments TC503 temperature controller for low temperature data collection. The main parameters used for computation were: (i) The average value of the hyperfine coupling constant, $<A>=(A_{xx}+A_{yy}+A_{zz})/3$, for the coupling between the electron spin and the nitrogen nuclear spin; this parameter provides a measure of the environmental polarity of the probe. (ii) The correlation time for the diffusional rotational motion of the probe, τ, which measures the microviscosity at the probe site, in turn related to the probe-surface interactions. (iii) The line width, LW, measuring the spin-spin interactions between paramagnetic moieties sitting in close positions. (iv) In case the probes concentrate in a restricted space (as such as hydrogel interstices), fast collisions between probes lead to a high exchange frequency, W_{ex}, which therefore accounts of the presence of concentrated pools of probe solutions into hydrogel interstices.

Molecular Dynamics (MD) Simulations. All MD simulations were performed using GROMACS-2018[2] on initial structures generated through Molecular Operating Environment (MOE).[3] The initial structures were generated to capture the hydrogel cores of **H3** and **H6**, as shown in the Figure S.9. These simulations were used to compare the hydration of the hydrogels. Two additional sets of simulations were performed for both systems containing the drug molecules, Ibuprofen and Curcumin, respectively, which

were introduced in proximity to the hydrogels to assess their interactions with the hydrogel core.

All systems were simulated in a 3D box (~ 6x5x5 nm^3) containing the hydrogel core, ~ 4000 water molecules and monovalent Na and Cl counterions. The system was subjected to energy minimization to prevent any overlap of atoms, followed by a 5 ns equilibration run. The equilibrated system is followed by a production run of 100 nanoseconds. The MD simulations incorporated leap-frog algorithm with a 2fs time-step to integrate the equations of motion. The system was maintained at 300K and 1 bar, using the velocity rescaling thermostat[4] and Parrinello-Rahman barostat[5], respectively. The long-ranged electrostatic interactions were calculated using particle mesh Ewald (PME)[6] algorithm with a real space cut-off of 1.2 nm. LJ interactions were also truncated at 1.2 nm. TIP3P model[7] was used represent the water molecules, and LINCS[8] algorithm was used to constrain the motion of hydrogen atoms bonded to heavy atoms. Coordinates were stored every 10 ps for further analysis. The RMSD and number of contacts were calculated using gromacs subroutines, rms & mindist. The number of hydration waters and hydrogen bonds were calculated using Visual Molecular Dynamics (VMD).[9]

General MTT assay protocol. Cell viability was assessed by MTT (3-(4,5-dimethylthiazol-2-yl)-2,5-diphenyltetrazolium bromide) assay. PC-3 cells were seeded in monolayers ($5x10^3$ cells/well) in 96-well plates (Surface Delta Nunclon, Thermo Fischer Scientific) and cultured in complete medium for 24 h. Cells were cultured with media containing the drug and incubated for 24 h. After this incubation time, MTT solution (0.3 mg/mL) was added to the cell plates and incubated for 1.5 h. Finally, the cell culture was removed and the purple formazan crystals were dissolved in DMSO. The optical density, directly proportional to the number of live cells, was quantified at 570 nm (with a background correction of 620 nm) using a multiwell plate reader.

Cytotoxicity assays.

(1) Hydrogels sterilization and loading. Pristine hydrogels **H3**-**H8** hydrogels (32 mg in dynamic test, 14.5 mg in static test) were sterilized through UV irradiation for 2 h. Selected hydrogels were then immersed in 1 mL of a 10 mg/mL solution of curcumin in ethanol, shaking at 150 rpm for 30 min. Afterwards, they were dried under vacuum.

(2) Leach-out ("static") assay and MTT evaluation.

Pristine or CUR-loaded hydrogels were immersed in complete culture medium (500 µL for pristine, 850 µL for CUR-loaded) and potential leachable substances were extracted at 37°C under orbital shaking (150 rpm). Leach out media was collected after 6 h (425 µL) and 24 h (425 µL) for loaded hydrogels, or at 24 h (500 µL) for pristine hydrogels. PC-3 cells were seeded in monolayers ($5x10^3$ cells/well) in 96-well plates (Surface Delta Nunclon, Thermo Fischer Scientific) for 24 h and then exposed to the leach-out media for 24 h. Cell viability was then assessed through MTT assay.

(3) Trans-well ("dynamic") assay and MTT evaluation.

In 24-well plates with transwell, at different times (2, 6, 24 and 48 h) the hydrogels are introduced in the transwell together with 200 µL of medium (the hydrogel is submerged in the medium), first introducing the one that will be exposed to the cells for the longest time. The plate is placed in the incubator (37°C and static). After this time in which the gel is releasing the drug, a MTT solution was added and cytotoxicity was quantified as described in the general protocol.

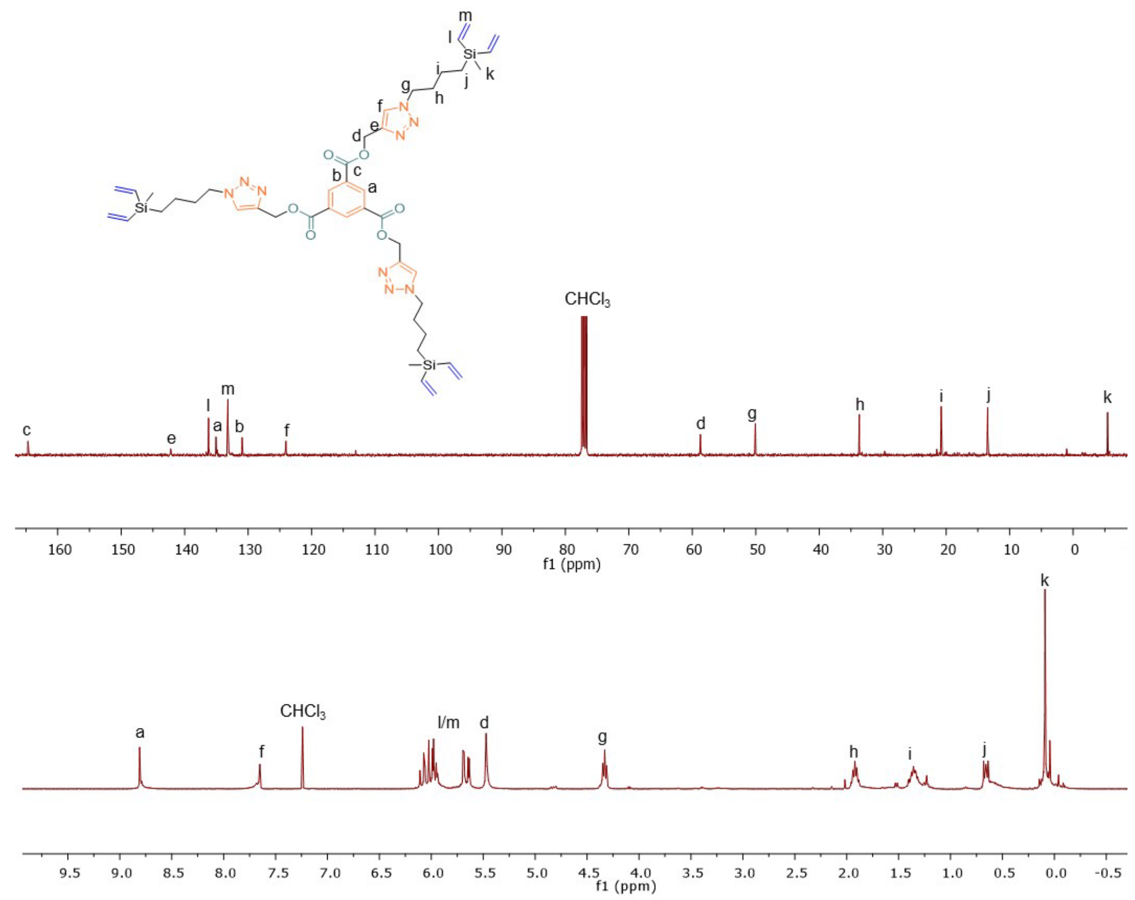

Figure S1. ^{1}H and ^{13}C NMR spectra of dendrimer ArG1V6 (**3**) in $CDCl_3$.

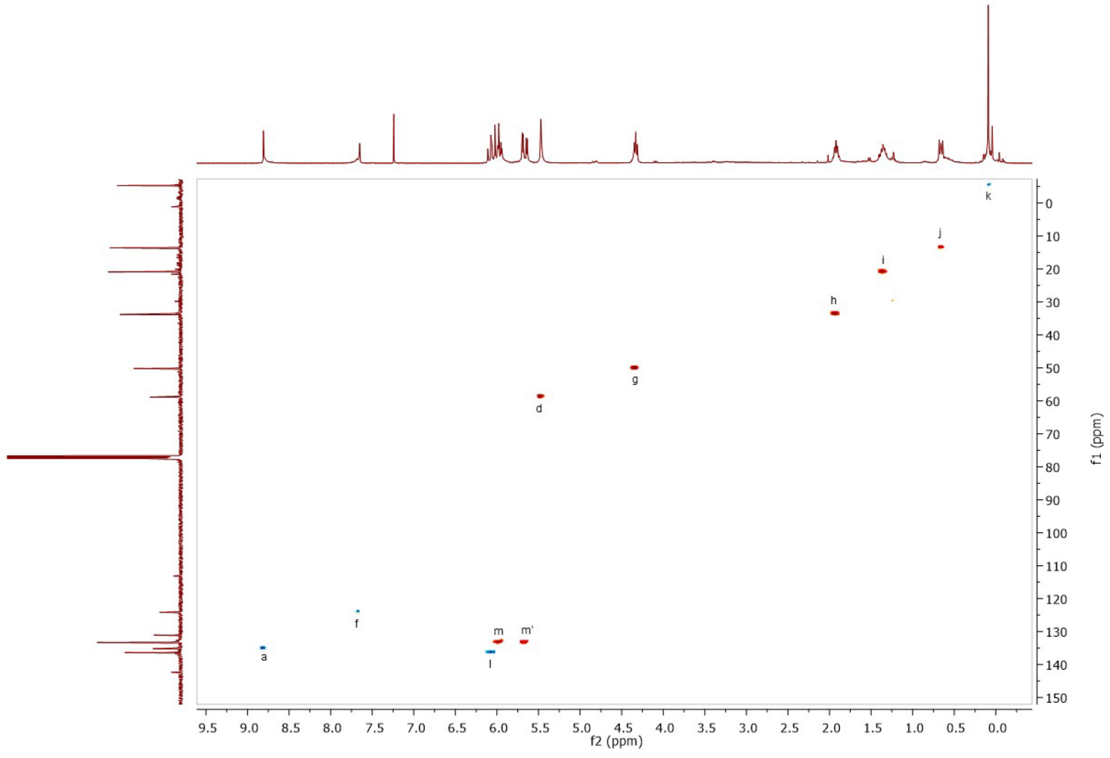

Figure S2. ^{1}H-^{13}C HSQC spectrum of dendrimer ArG1V6 (**3**) in $CDCl_3$.

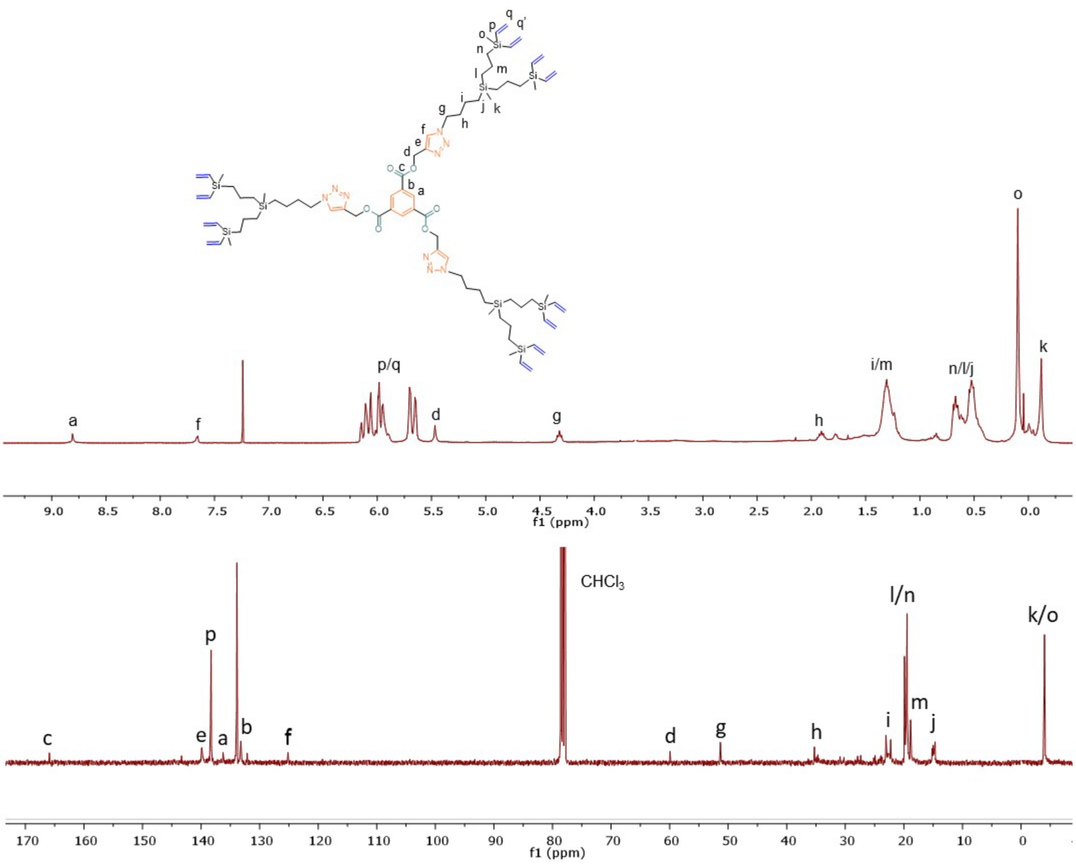

.

Figure S3. ^{1}H and ^{13}C NMR spectra of dendrimer ArG2V12 (**4**) in $CDCl_3$.

q q'

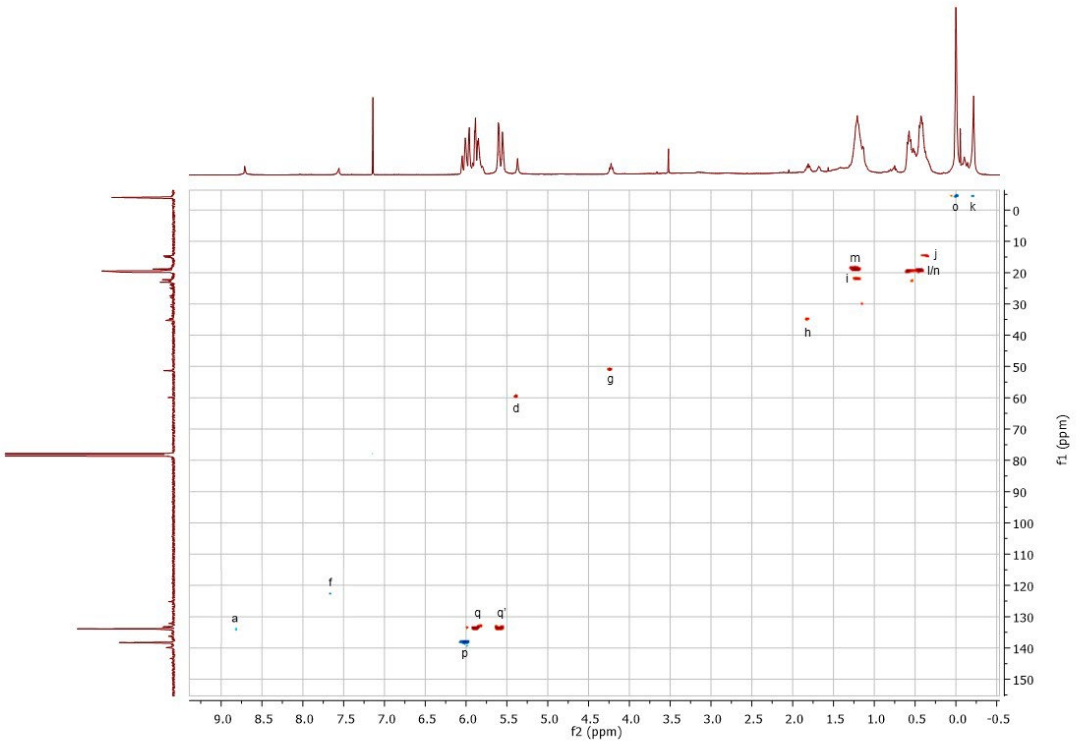

Figure S4. ^{1}H-^{13}C HSQC spectrum of dendrimer ArG2V12 (**4**) in $CDCl_3$.

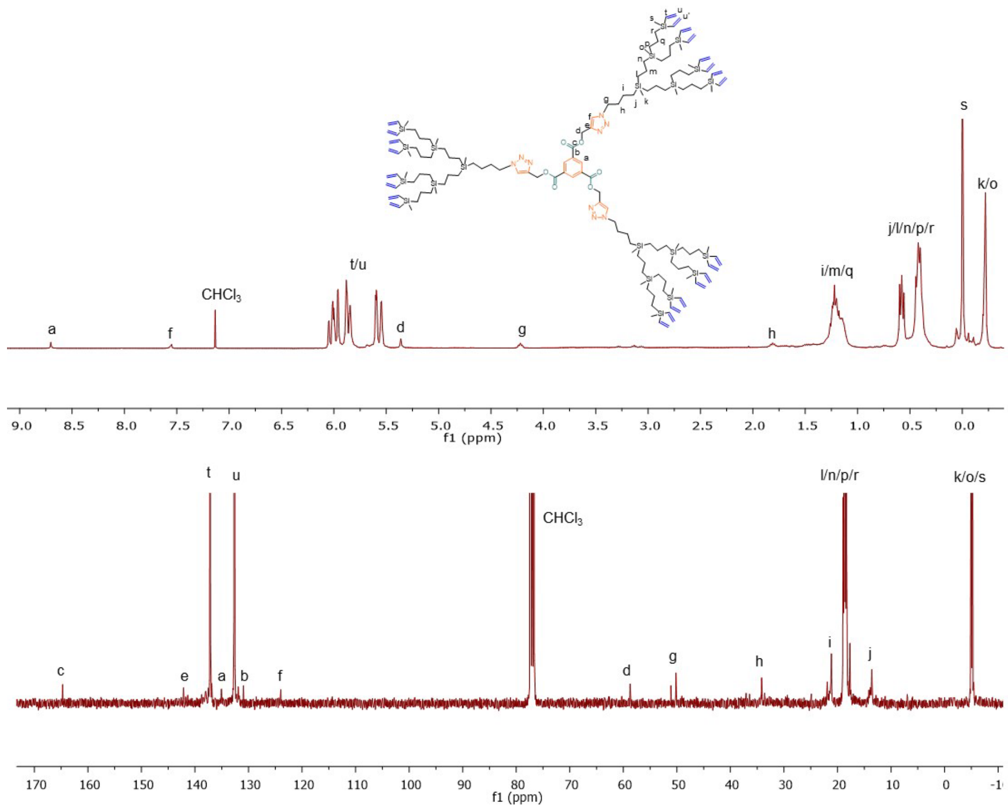

Figure S5. ^{1}H and ^{13}C spectra of dendrimer ArG3V24 (**5**) in $CDCl_3$. curves for hydrogels **H7-H8**.

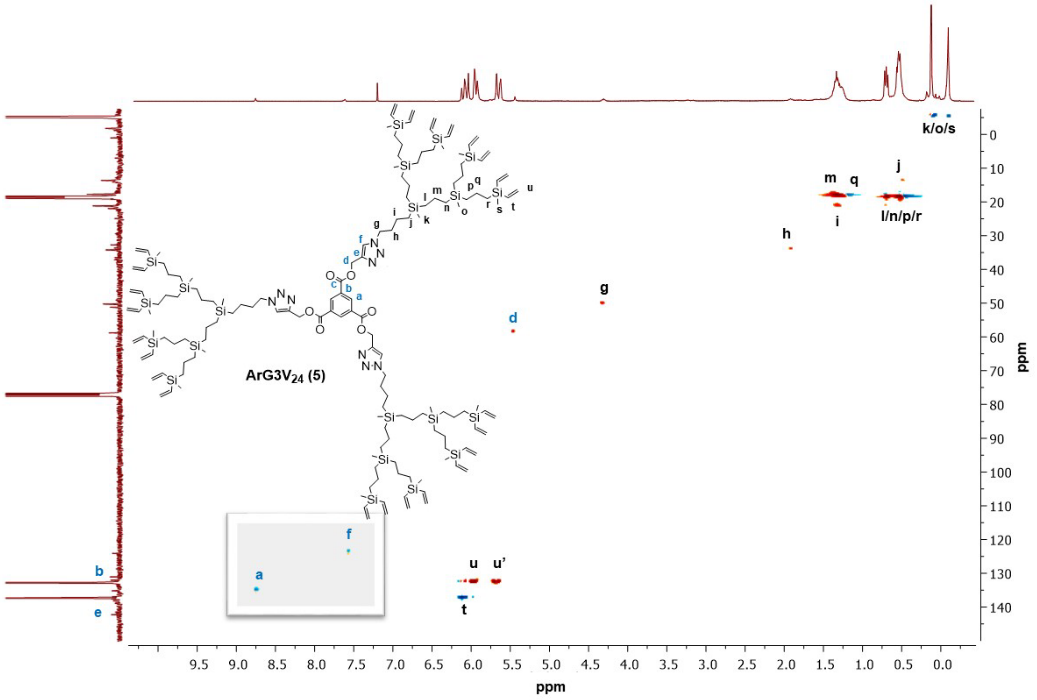

Figure S6. Edited ^{1}H-^{13}C HSQC spectrum at 400 MHz of aromatic dendrimer ArG3V$_{24}$ (**5**) in $CDCl_3$, including signal assignment. Spots from CH_2 groups are shown in red and CH/CH_3 appear in blue. Core signals are highlighted in blue.

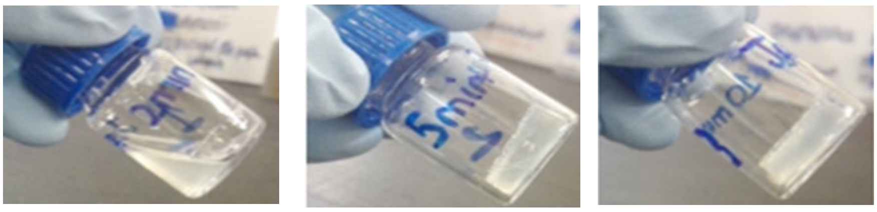

Figure S7. **H7** Hydrogel formation after 2, 5 and 10 min exposure to UV-light.

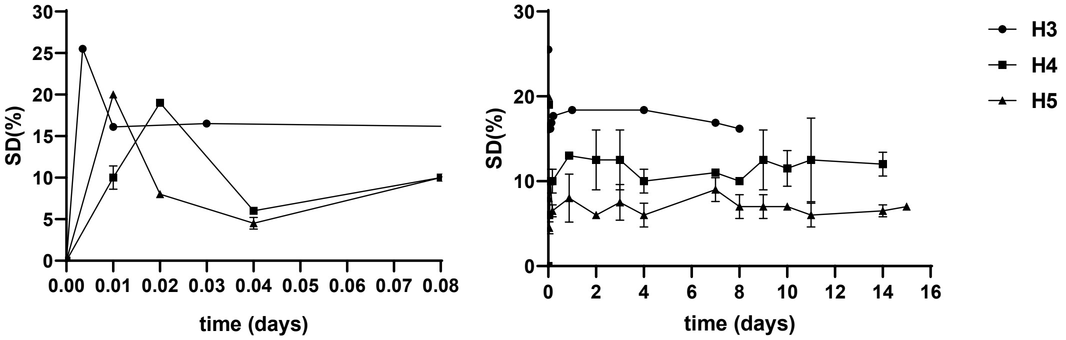

Figure S8. Swelling degree over time for aromatic-core hydrogels **H3, H4** and **H5**.

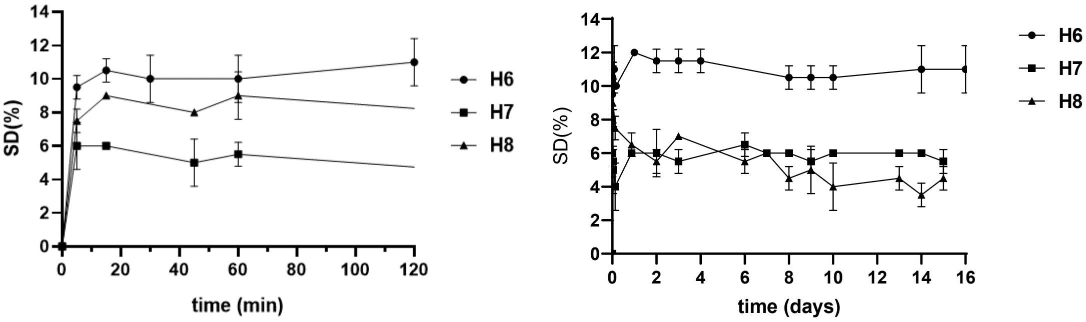

Figure S9. Swelling degree over time for Si-core hydrogels **H6**, **H7** and **H8**.

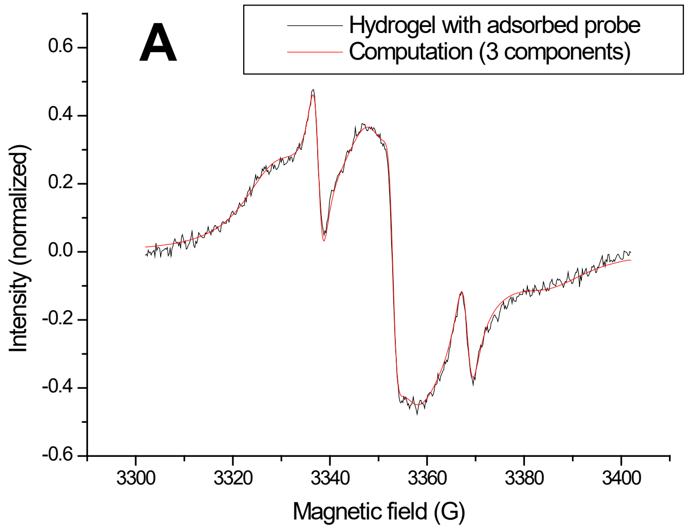

Figure S10. Experimental (black) and computed (red) EPR spectra of hydrogel **H3** loaded with the probe 4-Benzoyloxy-TEMPO.

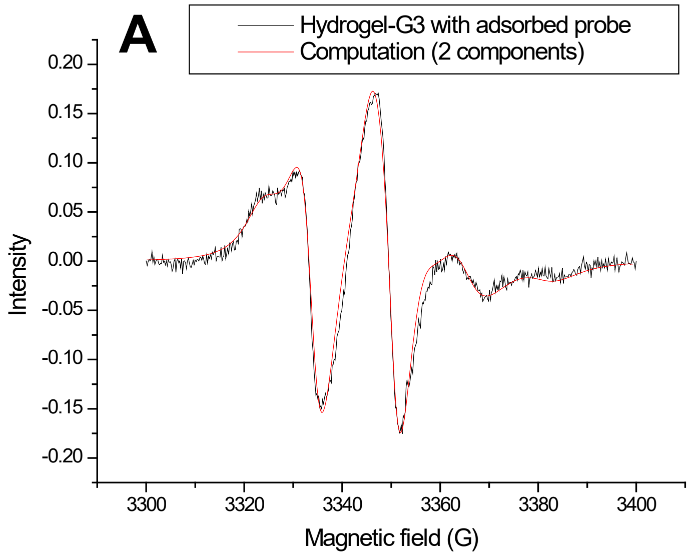

Figure S11. Experimental (black) and computed (red) EPR spectra of hydrogel **H5** loaded with the probe 4-Benzoyloxy-TEMPO.

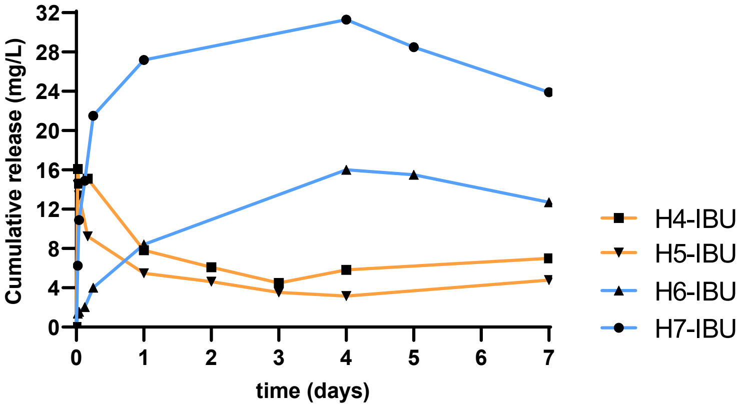

Figure S12. Ibuprofen-release curves for hydrogels **H4-H7**.

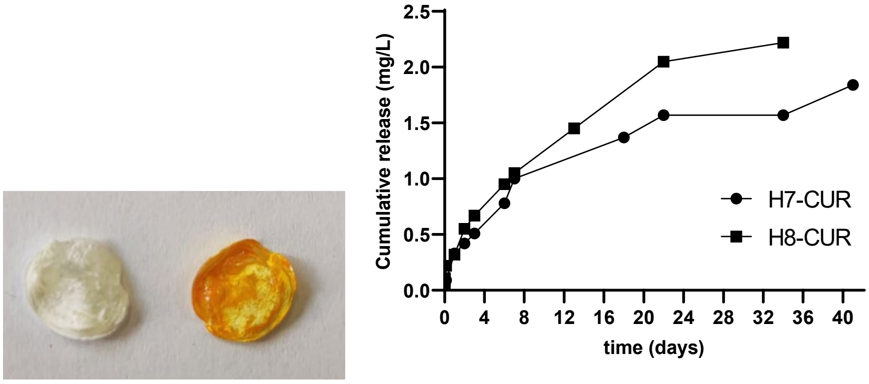

Figure S13. A. Aspect of a hydrogel before and after encapsulation of curcumin. B. Curcumin-release curves for hydrogels **H7-H8**.

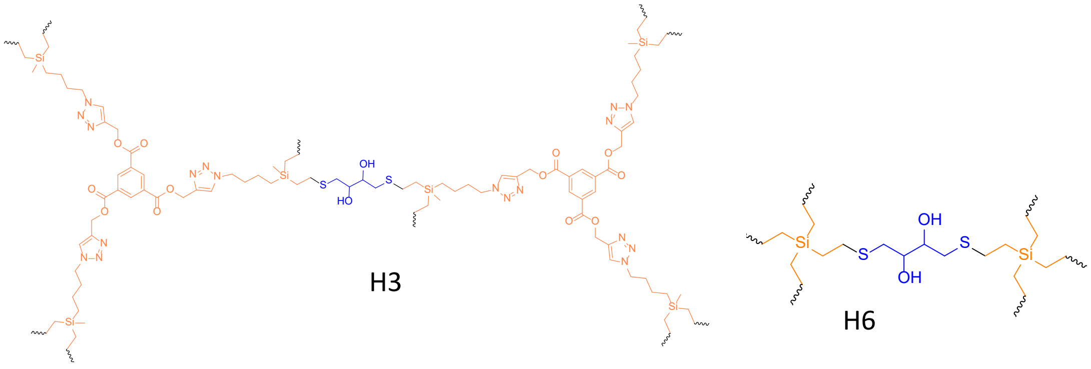

Figure S14. Selected crosslinking sections used in the Molecular Dynamics simulations.

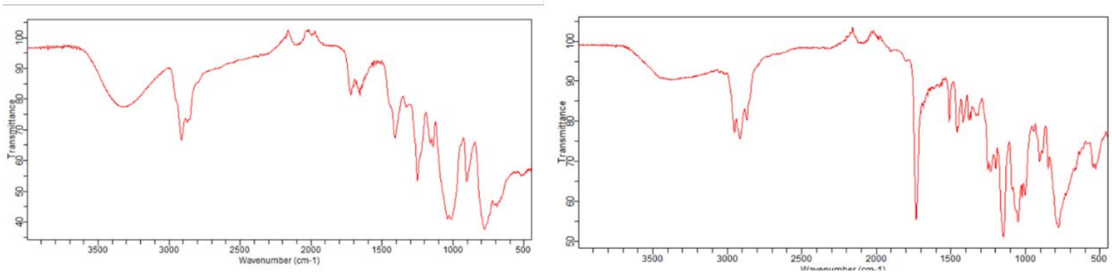

Figure S15. FTIR spectra of hydrogel **H4**, before (left) and after (right) esterification of ibuprofen.

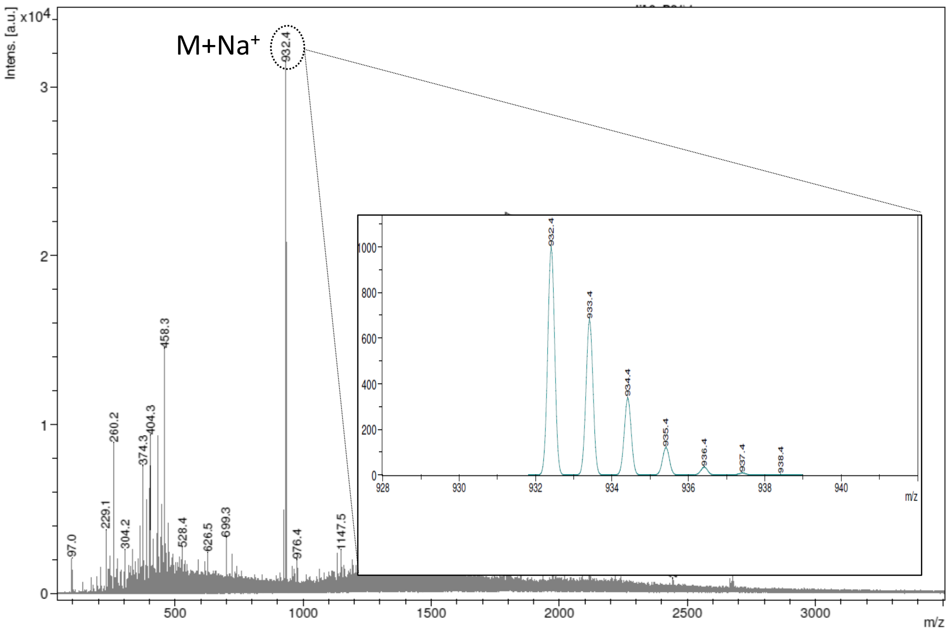

Figure S16. MALDI-TOF spectra dendrimer **3** in DCTB. Pred.: M = 909.42; obs.: M+Na$^+$ = 932.4.

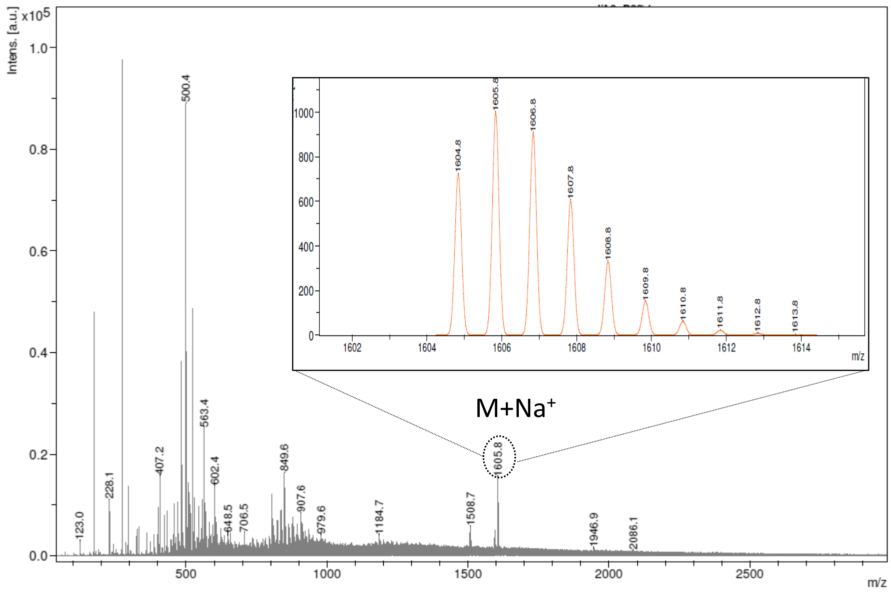

Figure S17. MALDI-TOF spectra of dendrimer **4** in DCTB. Predicted: M = 1582.85; observed: M+Na$^+$ = 1605.8.

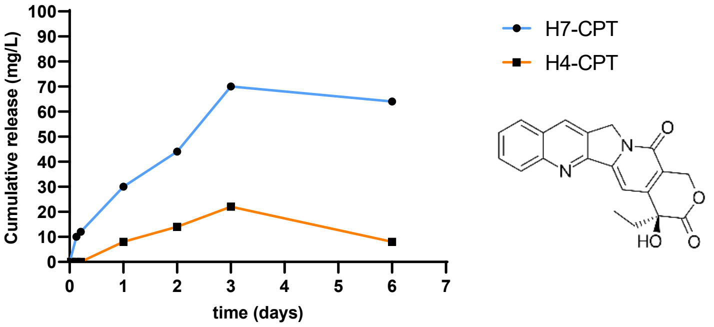

Figure S18. Captothecin-release curves for selected hydrogels **H4** and **H7**. For **H4**, 7.61 mg of hydrogel were used (with 0.9 mg of loaded drug); for **H7**, 24.8 mg of hydrogel were used (with 1.3 mg of loaded drug).

---

[1] E. Fuentes-Paniagua, C. E. Peña-González, M. Galán, R. Gómez, F. J. de la Mata and J. Sánchez-Nieves, *Organometallics*, 2013, **32**, 1789-1796.

[2] Abraham, M.J., Performance enhancements for GROMACS nonbonded interactions on BlueGene. J Comput Chem, 2011. 32(9): p. 2041-6.

[3] Molecular Operating Environment (MOE), C.C.G.U., 1010 Sherbooke St. West, Suite #910, Montreal, QC, Canada, H3A 2R7, 2021

[4] Bussi, G., Donadio, D. and Parrinello, M. (2007) Canonical sampling through velocity rescaling. Journal of Chemical Physics, 126.

[5] Berendsen, H.J.C., Postma, J.P.M., Vangunsteren, W.F., Dinola, A. and Haak, J.R. (1984) Molecular-Dynamics with Coupling to an External Bath. Journal of Chemical Physics, 81, 3684-3690.

[6] Darden, T., York, D. and Pedersen, L. (1993) Particle Mesh Ewald - an N.Log(N) Method for Ewald Sums in Large Systems. Journal of Chemical Physics, 98, 10089-10092.

[7] Jorgensen, W.L., Chandrasekhar, J., Madura, J.D., Impey, R.W. and Klein, M.L. (1983) Comparison of Simple Potential Functions for Simulating Liquid Water. Journal of Chemical Physics, 79, 926-935.

[8] Hess, B., Bekker, H., Berendsen, H.J.C. and Fraaije, J.G.E.M. (1997) LINCS: A linear constraint solver for molecular simulations. J Comput Chem, 18, 1463-1472.

[9] Humphrey, W., Dalke, A. and Schulten, K., "VMD - Visual Molecular Dynamics", J. Molec. Graphics, 1996, vol. 14, pp. 33-38
